# Rapid and sensitive detection of mycotoxins by advanced and emerging analytical methods: A review

**DOI:** 10.1002/fsn3.1474

**Published:** 2020-03-25

**Authors:** Jyoti Singh, Alka Mehta

**Affiliations:** ^1^ Department of Integrative Biology School of Biosciences and Technology Vellore Institute of Technology Vellore India

**Keywords:** advanced quantitative techniques, chromatography, immunological, mycotoxins, spectroscopy

## Abstract

Quantification of mycotoxins in foodstuffs is extremely difficult as a limited amount of toxins are known to be presented in the food samples. Mycotoxins are secondary toxic metabolites, made primarily by fungal species, contaminating feeds and foods. Due to the presence in globally used grains, it is an unpreventable problem that causes various acute and chronic impacts on human and animal health. Over the previous few years, however, progress has been made in mycotoxin analysis studies. Easier techniques of sample cleanup and advanced chromatographic approaches have been developed, primarily high‐performance liquid chromatography. Few extremely sophisticated and adaptable tools such as high‐resolution mass spectrometry and gas chromatography–tandem MS/MS have become more important. In addition, Immunoassay, Advanced quantitative techniques are now globally accepted for mycotoxin analysis. Thus, this review summarizes these traditional and highly advance methods and their characteristics for evaluating mycotoxins.

## Graphical abstract

Mycotoxin causes various acute and chronic impact on human and animal health. It is unavoidable contaminant in food and feed. Thus, this review summarizes these traditional and highly advance methods and their characteristics for evaluating mycotoxins.

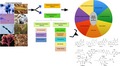

## 1 INTRODUCTION

Population in developing countries especially rural areas are dependent on locally produced foods and generally face problems related to food security and mycotoxin contamination which is reflected to be a major food quality issue. Mycotoxins are toxic, low‐molecular weight (300–700 Da), secondary fungal metabolites which are produced in both pre‐ and postharvest conditions. Their yield is genotypically identified, but is not narrowed to only one toxin per species or one species (Turner, Subrahmanyam, & Piletsky, 2009). The frequently monitored mycotoxins include aflatoxin, ochratoxin A, patulin (PAT), zearalenone, groups of trichothecenes, fumonisin, and citrinin (Lerda, 2011). The conventional analytical methods applied to determine mycotoxin in food samples produce outcomes within periods or days. The existing competition between the food and feed industry, thereby, drives to decrease costs, service, labors, and rapid delivery of results. Therefore, rapid techniques for the analysis of mycotoxin have become increasingly significant.

This review summarized a wide outline about the detection techniques, qualitative and quantitative determination methods of major and minor mycotoxins over the previous 50 years. A brief discussion about various mycotoxin sources has been included. Attention is drawn toward the toxicity of different mycotoxins and their metabolites as well as their adverse effects on human health. Precleaning methods play an important role in the detection of mycotoxins. Hence, this review provides a brief summary of several reported extraction methods and also on various advancements employed in solid‐phase extraction methods. In this review, various detection techniques viz. ultraviolet, fluorescence, photomultiplier, ion mobility, and tandem mass spectrophotometry, fourier transforms near infrared, adsorptive stripping voltammetry, and their lower detection limits, and sensitivity of different types of matrice has been reviewed. For the determination of mycotoxins, traditional quantitative methods viz. chromatography, immunological, and the advanced methods viz. ultrahigh‐performance liquid chromatography, fluorescence polarization immunoassay, nanoparticle‐based methods, microfluidics, and phage display methods have been discussed extensively in this review.

### 1.1 Occurrence of major mycotoxins

#### 1.1.1 Aflatoxin

**Figure 1:**
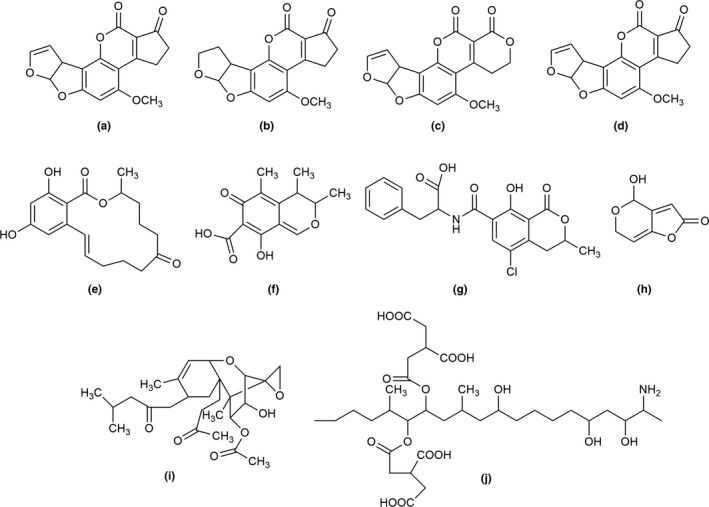
Chemical structures of the mycotoxins abbreviations: (a) Aflatoxin B_1,_ (b) Aflatoxin B_2,_ (c) Aflatoxin G_1,_ (d) Aflatoxin G_2,_ (e) Zearalenone, (f) Citrinin, (g) Ochratoxin, (h) Patulin, (i) Trichothecenes, (j) Fumonisin B_1_

Aflatoxins (AFs) are difuranocoumarins derivatives (Figure 1a‒d) produced by several strains of *Aspergillus parasiticus* and *Aspergillus flavus* via polyketide pathway (Alcaide‐Molina, Ruiz‐Jiménez, Mata‐Granados, & Luque de Castro, 2009; Ali et al., 2005). The four important AFs found are Aflatoxin B_1_ (AFB_1_), Aflatoxin B_2_ (AFB_2_), Aflatoxin G_1_ (AFG_1_), and Aflatoxin G_2_ (AFG_2_) and can be differentiated according to their fluorescence under UV light (green or blue) and comparative chromatographic movement during thin‐layer chromatography. Apart from major AFs, AFM_1_, a hydroxylated metabolite of AFB_1,_ frequently found in milk and milk based baby foods.

#### 1.1.2 Zearalenone

Various *Fusarium* species like *Fusarium graminearum*, *F. culmorum*, *F. equiseti*, and *F. crookwellense* are in the production of nonsteroidal estrogenic mycotoxin named zearalenone (Figure 1e; Urry, Wehrmeister, Hodge, & Hidy, 1966) via polyketide pathway (Hagler, Towers, Mirocha, Eppley, & Bryden, 2001). It exhibits blue‐green fluorescence when excited by long wavelength UV light (360 nm) and a more intense green fluorescence when excited with short wavelength UV light (260 nm; Yu, Wang, & Sun, 2014). Other techniques like HPLC/IAC, atmospheric pressure chemical ionization (APCI) or electrospray ionization interface and LC‐MS/MS have been commonly used for the measurement of zearalenone (ZEA; Berthiller, Schuhmacher, Buttinger, & Krska, 2005; Macdonald et al., 2005).

#### 1.1.3 Citrinin

Several species of *Aspergillus*, *Monascus,* and *Penicillium* are responsible for the production of Citrinin (Figure 1f). Among *Aspergillus* species, *A. niger* is reported to be mainly involved in the production of citrinin. Citrinin is a polyketide mycotoxin. It has a conjugated, planar structure which produces its natural fluorescence (the highest fluorescence is produced by a nonionized citrinin molecule at pH 2.5; Vazquez et al., 1996). Quantitative methods such as high‐performance liquid chromatography with fluorescence detection (HPLC‐FLD) and LC‐MS/MS have been compared for citrinin detection in red fermented rice samples, and it was observed that LC‐MS/MS displayed better results in terms of limit of detection (LOD) and quantification compared to that of HPLC‐FLD (Ji et al., 2015).

#### 1.1.4 Ochratoxin

Filamentous species of *Penicillium* and *Aspergillus* are involved in the production of Ochratoxin A (OTA; Figure 1g; Bredenkamp, Dillen, Rooyen, & Steyn, 1989; Budavari, 1989; Miller, 1992). It is a pentaketide derivative coupled to β‐phenylalanine from the dihydrocoumarins family. OTA is optically active, and it is spectrally characterized by UV‐visible, fluorescence, IR, and NMR and MS detection methods (Abramson, 1987; de Jesus, Steyn, Vleggaar, & Wessels, 1980).

#### 1.1.5 Patulin

Several species of mold, like *Penicillium*, *Aspergillus,* and *Byssochlamys,* are involved in PAT production. Patulin (Figure 1h) is also a polyketide metabolite. Liquid chromatography (LC) with UV detector has been used to identify and quantify PAT. However, capillary micellar electrokinetic chromatography (MEKC) developed by Tsao and Zhou (2000) has proved to be a faster and more precise technique for quantification of PAT. Martin, Aranda, Benito, Perez‐Nevado, and Cordoba (2005) have reported detection of five other mycotoxins such as citrinin, ZEA, mycophenolic acid, aflatoxin B_1,_ and griseofulvin apart from PAT by MEKC. It requires a small volume of samples and is ecologically safe compared to other analytical methods.

#### 1.1.6 Trichothecenes

Trichothecenes (Figure 1i) include a large family of structurally related toxins, mainly produced by fungi belonging to the genus *Fusarium*. Among trichothecenes, type A and type B are of special interest due to their widespread presence and extremely toxic nature. The trichothecenes contain a family of closely related chemical compounds called sesquiterpenoids. Derivatization using fluorescence labeling reagents, like 1‐Anthroylnitrile (1‐AN), is an effective method for determination of trichothecenes (Pascale, Haidukowski, & Visconti, 2003; Trebstein, Seefelder, Lauber, & Humpf, 2008; Visconti, Lattanzio, Pascale, & Haidukowski, 2005). Methods like GC–flame ionization detector (FID)/mass spectrophotometer (MS) and enzyme‐linked immunosorbent assay (ELISA) have been used for separation, identification, and quantification of trichothecenes (Li et al., 2012; Turner et al., 2009). ELISA proved to be a cheap and rapid screening method among others.

#### 1.1.7 Deoxynivalenol and deoxynivalenol‐3‐glucoside

Deoxynivalenol (DON) is a major trichothecenes, one of several *fusarium* species mycotoxins. Maize, wheat, oats, barley, rice, and other grains are often contaminated in the field or during processing. DON can be converted to deoxynivalenol‐3‐glucoside (DON‐3G) called as masked mycotoxin by plant detoxification (Dong et al., 2017). Methods like LC‐MS/MS are developed to detect the both DON and DON‐3G in the bakery products (Generotti et al., 2015). Similarly, Johny et al. (2019) have developed high‐resolution LC‐MS method to detect DON‐3G exposed fish and in plant‐based fish feed. The LOD was obtained 176 µg/kg for DON‐3G in salmon, zebrafish, and fish feed.

#### 1.1.8 Fumonisin

Fumonisin (Figure 1j) is produced by *Fusarium* species particularly *Fusarium proliferatum*, *F. nygamai*, *F. verticillioides*, and *Alternaria alternate* (Rheeder, Marasas, & Vismer, 2002). Plattner and Shackelford (1992) and Seo and Lee (1999) have demonstrated that fumonisins (FBs) do not possess a cyclic structure which is generally found in mycotoxins. The detection and measurement of these toxins by HPLC using electrospray MS and evaporative light scattering detector have been widely reported. Fumonisins can be detected using HPLC‐UV or HPLC fluorescence detectors after derivatization (Ndube, van der Westhuizen, & Shephard, 2009; Shephard, Sydenham, Thiel, & Gelderblom, 1990).

### 1.2 Mycotoxins toxicity and their adverse effects

Mycotoxins are the most hazardous among food and feed contaminants, due to the global occurrence of mycotoxigenic molds. They have adverse effects on human and animal health. These toxic effects vary according to the chemical structure of the toxin. Not only the amount of the toxin but also the duration of exposure determines the degree of such adverse effects. International Agency for Research on Cancer (IARC) has categorized mycotoxins as proven (Group 1), probably (Group 2A) and possibly (Group 2B) human carcinogen. For instance, AFs have been categorized under Group1 and OTA under Group 2B (Bhat, Rai, & Karim, 2010). AFs are hepatotoxic and proven as a hepatocarcinogenic agents (Mishra & Das, 2003). AFs are immunosuppressive, teratogenic, and mutagenic in nature. Only Ochratoxin is potentially as important as AFs among the *Aspergillus* toxins. The main target organ is the kidney. OTA is a nephrotoxin for all animal species tested so far and is most likely to be toxic to humans (Creppy, 1999). It causes neurotoxicity and hepatotoxicity and affects blood coagulation and immunosuppressive carcinogenic agent. In all animal species studied, citrinin also acts as a nephrotoxin, but its acute toxicity varies among different species (Carlton & Tuite, 1977). It also causes hepatotoxicity and genotoxicity (Group 3, IARC). ZEA (group 3, IARC) has strong estrogenic effects, and trichothecenes (Group 3, IARC) can inhibit protein synthesis, induce immune‐modulatory effects, alimentary toxic aleukia (Sudakin, 2003). Patulin generally found in apples and in unfermented apple juice (Trucksess & Tang, 1999). In general, toxicity of PATs is related to acute and subacute toxicity, genotoxicity, embryotoxicity, and teratogenicity (Puel, Galtier, & Oswald, 2010).

The United States, the European Commission, and many other countries have established a tolerable daily intake and maximum residue levels (MRLs) for AFB_1_, OTA, FBs, ZEA, DON (Group 3), and trichothecene (T‐2, HT‐2) toxins in different foodstuffs. In case of AFs, the limit is set in the range of 2–4 μg/kg in cereals, dried products, and peanuts (European Commission (EC), 2010). For FBs and OTA, the limit has been set to 200–1,000 μg/kg in cereals and cereal‐based products, and 2–10 μg/kg in cereals, wine, coffee, cheese, and cocoa, respectively. ZEA limits range from 20 to 100 μg/kg in cereals and cereal products. For DON and PAT have regulatory limits 200–500 μg/kg in cereals and cereal products and 10–50 μg/kg in apple and concentrate, respectively. Food and Drug Administration (FDA) has set the regulatory guidelines for major mycotoxins in food and feed. The United States has set MRLs as 20 μg/kg AFs in different food commodities like maize, wheat, rice, and peanut, 0.5 μg/kg AFM_1_ in milk and milk products, 2,000–4,000 μg/kg FBs in maize and maize products, 1,000 μg/kg DON in cereals and cereal products, and 50 μg/kg PAT in apple and apple juices (FDA, 2002). Due to the common occurrence of regulated mycotoxins (AFs, ZEA, DON, FBs, OTA), their toxic nature has posed a risk to human and animal health, therefore demanding a solution for the protection of fauna. Although some toxins have not been regulated such as alternaria, sporidesmins, endophyte mycotoxins, sterigmatocystin, and phomposins, their toxigenic potential has been assessed in various studies (Nieto, Granero, Zon, & Fernández, 2018; Rodríguez‐Carrasco, Moltó, Berrada, & Mañes, 2014; Woudenberg, Groenewald, Binder, & Crous, 2013). Regulated and unregulated mycotoxins and their toxicity were summarized in (Tables 1 and 2).

**Table 1:** Regulated mycotoxins and their toxicity

Regulated mycotoxins	Source	Toxicity	References
AFs (AFB_1,_ AFB_2_, AFG_1_, AFG_2_, AFM_1_, AFM_2_)	*Aspergillus flavus*, *A. parasiticus*, *A. bombyci*, *A. ochraceoroseus*, *A. nomius*, *A. pseudotamari*	Carcinogenic, teratogenicity, hepatotoxic mutagenic, nephrotoxic, liver disease and immunosuppressive, formation of DNA adducts, lipid peroxidation, bioactivation by cytochromes P450, conjugation to GS‐transferases	Mishra and Das (2003) and Streit et al. (2012)
FBs (FB_1_, FB_2_, FB_3_)	*Alternaria alternata, Fusarium anthophilum, F. moniliforme, F. dlamini, F. proliferatum, F. nygamai, F. verticillioides*	Carcinogenic, hepatotoxic, necrosis, immunotoxic, adverse effect on the sphinganin/sphingosin ratio, adverse effects on the cell cycle	Rheeder et al. (2002)
Type A trichothecenes (T­2 and HT­2 toxin, diacetoxyscirpenol, neosolaniol)	*Fusarium sporotrichioides*, *F. graminearum*, *F. moniliforme*, *F. myrothecium*, *F. acuminatum*, *F. culmorum*, *F. equiseti*, *Cephalosporium* sp.* Trichoderma* sp.	Immunodepressants, gastrointestinal, mutagenic induction of apoptosis in haemopoietic progenitor cells, effect on protein synthesis, abnormal changes to immunoglobulins.	Ueno (1984)
Type B trichothecenes (nivalenol, deoxynivalenol, 3­acetyl DON, 15­acetyl DON, fusarenon X)	*Fusarium graminearum*, *F. culmorum*, *F. sporotrichioides*, *F. cerealis*, *F. lunulosporum*	Immunodepressants, neurotoxic, mutagenic, gastrointestinal.	Zain (2011) and Ueno (1984)
ZEA	*Fusarium graminearum*, *F. culmorum*, *F. equiseti*, *F. sporotrichioides*	Estrogenic activity (infertility, vulvar edema, vaginal prolapse, hypertrophy in females, feminization of males), bioactivation by reductases.	Tang et al. (2014) and Zain (2011)
Ochratoxins (OTA, OTB, OTC)	*Aspergillus ochraceus*, *A. pseudoelegans*, *A. alutaceus*, *A. alliaceus*, *A. auricomus*, *A. glaucus*, *A. niger*, *A. carbonarius*, *A. melleus*, *A. albertensis*, *A. citricus*, *A. flocculosus.*	Carcinogenic (urinary tract, tumors), mutagenic, nephrotoxic, hepatotoxic teratogenic, effect on protein synthesis, inhibition of ATP production, detoxification by peptidases	Bhat et al.(2010) and Zinedine et al. (2007)
Patulin	*Asregillus clavatus*, *A. longivesica*, *A. terreus*, *Penicillium. expansum*, *P. griseofulvum*, *Byssochlamys *sp.	Immunodepressant, pulmonary and cerebral edema, nausea, gastritis, paralysis, convulsions, capillary damage, carcinogenic, indirect enzyme inhibition	Puel et al. (2010)
Ergot alkaloids	*Claviceps purpurea*, *C. fusiformis*	Effects on gastrointestinal, the central nervous system	Bennett and Klich (2003)
Citrinin	*Aspergillus terreus*, *A. carneus*, *A. niveus*, *Penicillium verrucosum*, *P. citrinum*, *P. expansum*	Nephropathy, yellow rice disease carcinogenic, cytotoxic effects	Bennett and Klich (2003) and Magro et al. (2016)

**Table 2:** Other mycotoxins and their toxicity

Unregulated mycotoxins	Source	Toxicity	References
Alternaria Mycotoxins (altenuene, alternariol, alternariol Monomethyl ether, Altertoxin I, altertoxin II	*Alternaria alternata*, *A. dauci*, *A. cucumerina*, *A. solani*, *A. tenuissima*, *A. citri*	Teratogenic, mutagenic, carcinogenic, cytotoxic effects.	Pedras, Zaharia, and Ward (2002)
Tremorgenic mycotoxins (penitrems, janthitrems, lolitrems, aflatrem)	*Penicillium roquefortii*, *P. crustosum*, *P. puberrelum*, *Aspergillus clavatus*, *A. fumigatus*	Gastroenteritis, neurotoxicity, clinically affected dogs present with cute abdominal pain, muscle tremors and fasciculation, ptyalism, vomiting, fever, tachycardia, hyperesthesia, and seizures.	Hocking, Holds, and Tobin (1988)
Sterigmatocystin	*Aspergillus nidulans*, *A. versicolor*, *A. flavus*	The toxic effects of sterigmatocystin are much the same as those of AFsAFB1.	Holzapfel, Purchase, Steyn, and Gouws (1966)
Sporidesmins	*Pithomyces chartarum*	Facial eczema in ruminants.	Munday (1985)
Stachybotryotoxins	*Strachybotrys chartarum*	Pulmonary hemorrhage, inhalation or contact exposure in human beings responsible for production losses in affected animals.	Etzel et al. (1998)
Phomopsins	*Phomopsis leptostromiformis*	Inhibition of important cellular functions such as spindle formation during mitosis and the intracellular transport of lipids. Distortions of cell nucleus shape plus apparent disruptions to membrane systems within the cell.	Battilani et al. (2011)

For better understanding of the global effect of mycotoxin contamination, accurate, more rapid, and highly sensitive methods are essential for routine identification and detection of these compounds. The diverse nature of the matrice, target, environment, time requirements, detection levels, and accessibility of appropriate technology are considered to be challenging. For developing an effective, precise, and sensitive method for mycotoxins analysis, a great deal of attention should be paid to the matrice effect. The matrice effect is the combined effect of all sample components other than an analyte of interest on quantification. If a particular element can be defined as having an effect, it is called interference. The matrice effect can be observed as a loss or increase in response and therefore results in estimation or overestimation of mycotoxin. The matrice effect therefore affects the precision, accuracy, and sensitivity of the analytical method. For mycotoxin analysis, the matrice effect is very important, since mycotoxins, themselves, are of various chemical entities and present in various sample matrices.

Thus, investigation and detection of mycotoxin contamination in foods and feeds have been a vital center of international and national activities over the years. For accurate and rapid determination of these mycotoxins in unprocessed cereals and cereal‐based products, sensitive, analytical methods are highly relevant to the toxicological implications to animals and humans and highly desirable in order to measure risk of exposure, further to confirm regulatory levels fixed by the United States, European Union, or different international organizations. Analysis of mycotoxins usually requires toxin extraction from the matrice with a suitable extraction solvent, a cleanup procedure in order to remove interfering elements from the extract, and lastly, determination/detection of the toxin by appropriate analytical instrumentation.

## 2 EXTRACTION AND PRECLEANING METHODS

Primary extraction (Pascale, 2009) is necessary for the determination of mycotoxins from the different matrices like (wheat, maize, peanut, etc.). Selection of a suitable solvent is required for all the extraction procedures (Gilbert & Vargas, 2003). The choice of solvent primarily depends on the type of analyte. Generally determination of mycotoxins from solid feeds and food requires organic solvents. The two recommended methods are solvent extraction and solid‐phase extraction (SPE).

### 2.1 Solvent extraction method

Solvent extraction is a process to distinguish compounds based on their relative solubility in two different immiscible liquids, usually an organic solvent and water. Solvents possessing low dielectric constants (tendency to be immiscible with water) are good at extracting nonpolar compounds for example mycotoxins. To decrease the respective miscibility, appropriate solvents such as acetonitrile or methanol must be mixed with water in the presence of salts. The polar analytes selectively move into the polar organic phase from the aqueous phase. The following factors like polarity, solvent power (selectivity), and reactivity should be considered while selecting a particular solvent system. The main disadvantage of the solvent extraction method is poor selectivity of most solvents, and the final extract obtained is often colored and viscous.

### 2.2 Solid‐phase extraction method

Solid‐phase extraction is considered to be significant for sample preparation in mycotoxins analysis. The application of SPE is basically determined by the sorbent consumed in the extraction column. Currently, an enormous number of solvents are accessible, and the commonly used group of sorbents include polymers, porous/graphitized Carbon, chemically modified silica gel, and selective sorbents (immunosorbents, molecularly imprinted polymers). Most preferably used selective solid phase is those dependent on immunoaffinity recognition, where the target mycotoxin acts as an antigen, and solid phase possesses a targeted antibody. The choice of pretreatment method depends on many parameters such as the availability of analytical instrumentation, template, and target. Mycotoxins are small organic molecules which have different solubility in different solvents, so further cleanup methods are required.

### 2.3 Immunoaffinity column

Immunoaffinity column (IAC), a system based on antigen–antibody interaction, has some advantages, including limited mycotoxin loss and total removal of interfering substances. Therefore, the use of IAC as a cleanup technique could greatly improve the accuracy of subsequent analysis compared to SPE extraction,. The formulation of specific antibody solid‐phase materials plays an important role in extraction procedure (Şenyuva & Gilbert, 2010; Tessini et al., 2010). Ma et al. (2013) concentrated on the development of immunoaffinity column using 1C11 antibody for the extraction of four different AFs from different kind of matrices. O'Riordan and Wilkinson (2009) accomplished an assessment of IA methods, linked with detection techniques (ELISA and HPLC‐FD) with or without postcleanup derivatization of the chili sample. The outcomes from this experiment favored HPLC performs better as a postcleanup quantification technique.

### 2.4 New absorbents

Many advanced nanomaterials, including carbon nanomaterials and magnetic carbon nanomaterials, have been used for mycotoxin determination. The main advantage of carbon nanomaterials is due to their high adsorption ability (Wang, Liu, Lu, & Qu, 2014). Graphene oxide (GO) and multi‐walled carbon nanotubes (MWCNTs) are the example of Nanomaterial used as absorbent. GO was recently used in preconcentration of the extraction of AFs from traditional Chinese proprietary medicines for the first time (Ran, Chen, Ma, & Jiang 2017). MWCNTs have been shown to adsorb type A trichothecenes and were therefore used as SPE sorbents in maize, wheat, and rice to purify and enrich mycotoxins (Dong et al., 2015). However, only AFs, ZEA, and the four trichothecenes of type A (T‐2, HT‐2, DAS, and NEO) were studied. Many other types of mycotoxins are awaiting evaluation of the appropriate nanomaterials.

### 2.5 QuEChERS extraction method

Lehotay et al. (2007) reported QuEChERS (quick, easy, cheap, effective, rugged, and safe) solid‐phase extraction method to detect twenty pesticides in 3 matrices (grapes, lettuces, and oranges) at levels ranging from 10 to 1,000 ng/g. Azaiez, Giusti, Sagratini, Mañes, and Fernández‐Franzón (2014) also reported a method to detect mycotoxin in dried fruits using quenchers extraction. This method is preferred over other methods due to its ability of simultaneous extraction of multiple mycotoxins, less solvent utilization, cost‐effectiveness, quick, and lower detection limit than EU regulations.

## 3 DETECTION TECHNIQUES

UV absorbance and fluorescence characteristics of mycotoxins have been utilized for their detection and quantification. Various detectors, namely, UV, fluorescence, laser‐induced fluorescence (LIF), mass spectrometry (MS), and photomultipliers (PTM). have been used for quantitative determination of mycotoxins.

### 3.1 Ultraviolet absorption

Ultraviolet–visible spectroscopy is a type of absorption spectroscopy. It has been reported that all the AFs have a molar absorptivity of 20,000 cm^2^/mol exhibiting maximum absorption at 360 nm (Akbas & Ozdemir, 2006). Experimental data suggested that the detection limit of AFs can be improved by the selection of appropriate method for extraction and cleanup procedure (Ali et al., 2005; Göbel & Lusky, 2004). The sensitivity of UV system is not enough to detect AFs in trace levels (Alcaide‐Molina et al., 2009) since its limit of detection reaches up to only micro molar ranges (Couderc, Caussé, & Bayle, 1998). Hence, fluorescence (FL) techniques have gained more popularity for AFs detection.

### 3.2 Fluorescence

Fluorescence is an important parameter for the analysis and characterization of molecules that emit energy at specific wavelengths. It has been reported that almost every AF exhibits a maximum absorption at 360 nm (Akbas & Ozdemir, 2006). Different techniques for AFs detection associated with fluorescence are illustrated in (Table 3).

**Table 3:** Detection of Aflatoxin by different analytical methods

Toxin	Matrix	Analytical method	Detection method	Detection limit	Reference
AFM_1_	Milk	ELISA based on nanoparticles	UV absorbance	4–25 ng/L	Radoi, Targa, Prieto‐Simon, and Marty (2008)
AFB_1_	Corn	LC	FD	2.00–5.00 ng/g	Brera et al. (2007)
AFB_1_	Food	Surface‐enhanced Raman scattering (SERS)‐based immunoassay	Silica‐encapsulated hollow gold nanoparticles	0.1 ng/ml	Ko, Lee, and Choo (2015)
AFB_1_	Cigarette smoke	LC	MS	3.75 pg	Edinboro and Karnes (2005)
AFB_1_	Maize	Immunochromatographic Assay	Highly Luminescent Quantum Dot Beads	0.42 pg/ml	Ren et al. (2014)
AFs	Complex dietary product	HPLC	FD	1.6–5.2 mg/kg	Zahn et al. (2009)
AFs	Milk, egg, meat	HPLC	UV and FD	UV‐ 0.1 mg/kg	Herzallah (2009)
AFB_1_ and AFM_1_	Various foodstuffs	Indirect Competitive ELISA	UV absorbance	0.13–0.16 mg/L	Jiang et al. (2013)
AFB_1_	Peanuts	Competitive FLISA (quantum dot linked)	FD	0.016 ng/ml	Zhang et al. (2014)

#### 3.2.1 Fluorescence spectrophotometer

It has been used to analyze AFs in cereals, mainly in peanuts. The fluorometric method can quantify AFs from 5 to 5,000 μg/kg in less than 5 min (Herzallah, 2009). Fluorometric derivatization is required for better analysis of AFs for enhancement of their fluorescence. The detection limit for AFs in this case is also slightly higher than the limit set by EU (4 μg/kg). Urraca, Marazuela, and Moreno‐Bondi (2004) have reported a method to analyze ZEA and α‐zearalenol in wheat samples and swine feed. The LOD achieved was 6 ng/g for ZEA in wheat samples and swine feeds. For α‐zearalenol, LOD achieved was 3, 4 ng/g in wheat and swine feed, respectively. Fluorescence detection provides better accuracy and higher precision in the broad concentration range.

#### 3.2.2 High‐performance liquid chromatography coupled with fluorescence detection

There is no doubt that fluorescence detectors are the most sensitive among all the advanced current HPLC sensors. It enables to detect the presence of even a single analyte molecule in the flow cell. Usually, the sensitivity of fluorescence is 10–1,000 times better than that of UV detector for higher UV absorbing materials due to which this technique is used regularly in the measurement of specific fluorescent compounds present in the samples.

Although aqueous mixtures significantly enhanced the native fluorescence emission of AFs in reversed‐phase chromatography, AFs might be identified in food commodities by HPLC‐FLD. HPLC‐FLD is mostly used for the detection of OTA in several matrices, such as rice (Zinedine et al., 2007) and blue cheese (Dall'Asta et al., 2008). Kong et al. (2013) have developed a method for analysis of seven mycotoxins (AFB_1_, AFB_2_, AFG_1_, AFG_2_, ZEA, α zearalenol, and β zearalenol) in coix (*Coix lacryma‐jobi*)seed. The technique is based on the use of methanol/water (80/20) for fast ultrasonic solid–liquid removal, followed with the cleanup of the IAC, photochemical derivatization, and HPLC‐FLD. The detection limit for mycotoxins ranged from 0.01 to 0.04 μg/kg, which was noted to be lower than the tolerance levels set by the European Union (EU). This approach offers many advantages over recent practices, including sensitive detection and rapid separation.

#### 3.2.3 High‐performance liquid chromatography coupled with photodiode array

To obtain spectral profiles from molecular mixtures or chromatographically isolated samples, diode array detectors (also referred to as a DAD detector or more precisely HPLC photodiode array [PDA] detector) are used. An HPLC PDA detector is combined with separation system elutes by molecular weight, hydrophobicity (reverse‐phase), or ionic load, making them important for HPLC. PDA detectors used to analyze the molecules in different conditions such as solids or static solutions, or in a flow cell, it provides low noise spectral analysis. Mochamad and Hermanto (2017)have reported HPLC‐UV‐PDA array for the detection of AFB_1_ in cattle feed supplements. Limit of quantification was obtained as 2.3 ng of analyte for 1 gram of feed samples. Comparison of standard methods in worldwide regulations has been demonstrated to detect up to 4 ng analytes in 1 g samples with the use of a fluid chromatography instrument (Taheri et al., 2012). Results suggested that the method described above was more resilient than other methods for mycotoxin detection.

#### 3.2.4 Laser‐induced fluorescence screening method

Laser‐induced fluorescence coupled with HPLC is a sensitive and powerful technique used to detect AFs at subpicogram levels. Aflatoxins eluting from reverse‐phase column passes through detection window in LIF detector. The fluorescence induced by the laser is detected (Alcaide‐Molina et al., 2009; Simeon et al., 2001). Further multiples fiber optic LIF has been developed for direct detection of AFs in kernels of maize and pistachio (Smeesters, Meulebroeck, Raeymaekers, & Thienpont, 2015; Wu & Xu, 2019).

However, LIF detection is a technique constrained to a restricted number of research laboratories as it requires the labeling of analytes with dyes, having wavelength similar to laser and the laser itself is considered to be expensive. Furthermore, the LIF can generate false results when the labeling reactions are not up to the mark (Lalljie & Sandra, 1995).

### 3.3 Photomultipliers

Since fluorescence detection technique is concised to fluorescence, there is a need for other methods to detect mycotoxins. Photomultipliers (PMTs) are based on flow through the detection system, highly sensitive and are suited for the measurement of bioluminescence, chemiluminescence, or ultralow fluorescence (FIAlab Instruments, PMT‐FL). PTMs are sensors that are user‐friendly, compact for quick detection of AFM_1_ at low concentrations without the requirement for preconcentration of the sample (Cucci, Mignani, Dall'Asta, Pela, & Dossena, 2007).

### 3.4 Ion‐mobility spectrometry

The ion‐mobility spectrometry is a technique used to label chemicals that depend on the velocity achieved by the gas‐phase ions in the presence of an electrical field. The working of ion‐mobility spectrometry (IMS) is similar to that of Fourier Transform Near Infrared (FT‐NIR). The advantages of IMS include low detection limit, simple, fast response and cost‐effective. Khalesi, Sheikh‐Zeinoddin, and Tabrizchi (2011) have used corona discharge IMS to determine OTA in the licorice root. A detection limit (LOD) of 0.01 ng was achieved. The new technique is reliable if the extract is subjected to a prior immunoaffinity cleanup and is valid for selection purposes. Righetti et al. (2018) have developed ion‐mobility application to detect different mycotoxins such as AFs, OTA, HT‐2, T‐2, ZEA, PAT, DON, and fumonisins in wheat, malt, maize, and rye. The tests demonstrated high reproducibility (relative standard deviation (RSD) < 2%) in various instrument conditions and were not influenced by complex sample matrices, showing a RSD < 0.9%. In traditional LC and gas chromatography (GC) mass spectrometry (MS) workflows, several advantages are attributed to IMS: (a) It reduces the background noises and provides higher sensitivity in terms of detection of mycotoxins. (b) This provides additional data on mass spectrum and retention time, the so‐called collision cross section, so that compounds can be detected with greater confidence in targeted or nontargeted approaches.

### 3.5 Mass spectrometry/Tandem mass spectrometry

Mass spectrometry is an analytical technique that sorts the ions depends on their mass to charge ratio and ionizes chemical species. Tandem mass spectrometry (MS/MS) has an advantage in chromatographic peak detection. Mass spectrometry performance can be improved by combining it with various separation techniques, such as liquid chromatography, ion mobility, and gas chromatography. Pallarés, Font, Mañes, and Ferrer (2017) have reported the analysis of multi‐mycotoxin (AFs, 3aDON, 15aDON, Nivalenol, HT‐2, T‐2, ZEA, OTA, Enniatin, and Beauvericin) using LC–MS/MS method with a dispersive liquid–liquid microextraction (DLLME) procedure in a tea beverages matrice. The results showed LODs in the range of 0.05–10 μg/L. The risk assessment research showed that people are not exposed to mycotoxins using tea beverages.

### 3.6 Fourier Transform Near Infrared spectrometry

This technique depends on the absorbance quantity of the light emitted by the sample whose wavelength differs in the range of Near Infrared (NIR). An Infrared spectrometer is used in the analysis of a compound where Infrared radiations covering a wide range of frequencies are passed through the sample, and the radiant energy absorbed by each type of bonds in the molecules is measured. A spectrum is then produced normally consisting of a plot wave number (cm^−1^) versus transmittance (%). This technique has been used for the detection of AFs (Tripathi& Mishra, 2009) using the standard reference molecules with calibration. Bozza et al. (2013) have reported detection of OTA in the green coffee beans matrice. The transmittance and reflectance data showed good results in terms of detection and quantification of OTA in fungal isolates by FT‐IR method. Levasseur‐Garcia (2018) has described the detection of *fusarium* mycotoxins such as DON, ZEA, FB_1_, and FB_2_ present in corn, wheat, and barley. Spectrometry methods are time‐consuming and costly compared to infrared methods which are simple, fast, nondestructive methods to detect mycotoxins. Successful model preparation is an important criteria that must be established, thus requiring little sample preparation and some well‐trained technicians. This method has certain benefits, such as high accuracy, precision, and prediction of physical and chemical properties from a single spectrum allowing multiple mycotoxins to be determined simultaneously by the usage of multivariate calibrations.

## 4 TRADITIONAL QUANTITATIVE METHODS

The commonly used chromatographic methods for mycotoxins determination in cereals include thin‐layer chromatography (TLC), high‐performance liquid chromatography (HPLC) coupled with ultraviolet (UV), fluorescence (FD), diode array (DAD) or MS detectors, gas chromatography (GC) coupled with flame ionization (FID), and electron capture (ECD) or MS detectors. In addition to the abovementioned methods, commercial immunometric assays, such as membrane‐based immunoassays or ELISAs, are commonly used for selection purpose.

### 4.1 Chromatography methods

Numerous chromatographic methods are available for the quantification of mycotoxins. Traditional TLC is considered as an effective screening method for mycotoxins and has gained great significance due to low cost, simple instruments and fluorescent spots under UV, though it has poor accuracy and low sensitivity, making quantification difficult. TLC is widely accepted as an approved reference method for the determination of AFs; it has been replaced with HPLC for quantitative analysis of mycotoxins. Caputo et al. (2014) reported the development in TLC detection for OTA analysis. It was noted that when 2 µl was dropped onto the TLC plate, and 0.2 µg of OTA could be detected. This method shows better sensitivity than UV lamp and shows limit of detection as like LC methods as less as parts per billion (μg/kg).

#### 4.1.1 Liquid chromatography

Liquid chromatography has been developed to overcome the limitations of TLC method like limited plate length, humidity and temperature effect as the separation takes place in an open system. LC is generally coupled with UV absorption, amperometric detection, and fluorescence detection stage (FLD) with precolumn or postcolumn derivatization. LC coupled with fluorescence stage utilizes the fluorescence properties of AFs in order to quantify them. It has been acknowledged that LC‐MS and LC‐FLD are the benchmark methods for the detection of mycotoxins (Cirlini, Dall'Asta, & Galaverna, 2012; Núñez, Gallart‐Ayala, Martins, & Lucci, 2012).

For the simultaneous determination of mycotoxins like DON, NIV, FB_1_, FB_2_, T‐2 and HT2, ZEA, and AFs, LC‐APCI‐MS/MS method has been developed using reversed‐phase SPE Oasis^®^ HLB columns for extract cleanup (Lattanzio, Solfrizzo, & Visconti, 2008). Nowadays, different extraction columns such as DLLME, liquid–liquid extraction, SPE, accelerated solvent extraction, solid‐phase matrice dispersion, and dilute and shooting approaches have been reported with the aim of reducing the matrice effects as much as possible by reducing the interference from the extraction step with LC‐MS/MS to analyze multi‐mycotoxins (Santis et al., 2017; Pallarés et al., 2017).

Sensitivity, precision, and accuracy of LC‐MS techniques may differ based on the mycotoxins, matrice, ionization technique, and sensitivity of the process used. LC‐MS often gives unsatisfactory results of quantitative measurement of mycotoxins due to ion suppression and matrice effects. Tandem mass spectrometry has been preferred over fluorescence as the detection method since it is capable of identifying various nonfluorescent and fluorescent toxins and is cost‐effective.

#### 4.1.2 High‐performance liquid chromatography and ultrahigh‐performance liquid chromatography (UHPLC)

High‐performance liquid chromatography (HPLC) has been evolved, since the late 1960s. HPLC is considered as the common chromatographic method with a wide range of detection approaches (Vail and Homann, 1990). 80% of the world's organic compounds such as nutritional fortifiers, vitamins, protein, health food efficacy composition. have been analyzed using this method (Torres‐Pacheco, 2011). The food quality evaluation via this process offers an accurate, alternative, and acceptable mode to create strategies and to estimate the status of AFs in contaminated foods. The generally used detectors for HPLC are ultraviolet (UV) and fluorescence detectors (FLD). UV detectors are used to identify the analytes measuring the sample's absorption of light at different wavelengths. In case of fluorescence detector, analytes are identified depends on the occurrence of a chromophore in the particles. A number of toxins possess natural fluorescence property (e.g., AFs) and can be detected directly by HPLC‐FLD. Different analytical methods for AFs detection are summarized in (Table 3). Iha, Barbosa, Heck, and Trucksess (2014) and Iha, Barbosa, Okada, and Trucksess (2011) detected OTA and AFM_1_ in human milks and dairy products using fluorescence method linked with HPLC. Electrochemical and fluorescence detection is the two sensitive detection modes applied for quantitative studies in HPLC. Sensitive intensities of these amalgam techniques are much superior than conventional fluorescence. Arroyo‐Manzanares, Gamiz‐Gracia, and Garcia‐Campana (2012) used HPLC linked laser‐induced fluorescence for OTA detection. A simple liquid–liquid microextraction was applied to concentrate the toxin at low concentration (ng/L)and to reduce solvent requirements. The major limitations of HPLC methods are portability and practical issues based on the matrice effect, sample type, sample preparation, and choice of calibration. Therefore, there is a need for further analytical methodologies.

#### 4.1.3 Gas chromatography–Mass spectrometry (GC‐MS)

GC mainly depends on differential partitioning of analytes between the two phases of sample analysis. The different chemical components in the sample will distribute themselves between the stationary phase and mobile phase. After the completion of the separation process, the detection of the volatile products is carried out using either mass spectrometer and an electron capture detector (ECD) or FID. Rodríguez‐Carrasco, Moltó, Mañes, and Berrada (2014) and Rodríguez‐Carrasco, Berrada, Font, and Mañes (2012) developed a GC‐MS technique for analysis of mycotoxins (ZEA, PAT, trichothecenes) in grain products and human urine. This technique has a high specificity and sensitivity for all mycotoxins that can be derivatized to a compound which is sufficiently volatile to be gas chromatographed. The main problems associated with GC analysis for mycotoxin detections are as follows: nonlinearity of calibration curves, reminiscence properties from earlier samples, drifting responses, weak fluorescent and absorption groups, column blockage, and the risk of contamination compared to HPLC and LC methods (Pettersson & Langseth, 2002).

### 4.2 Immunological methods

#### 4.2.1 ELISA

Since late 1970s, immunological assays, such as ELISA, have gained much popularity for mycotoxins screening. Mycotoxins extract can be analyzed directly in the ELISA assay and does not require cleanup procedures. Such immunoassays provide fast, economical measurements even though the number of matrices tested are limited and often lack precision at low concentrations. Nevertheless, the occurrence of structurally related mycotoxins or matrice interference can obstruct conjugate and antibody binding vulnerable to errors in quantifiable mycotoxin measurements. Tang et al. (2014) validated an indirect ELISA detection technique with an immunoaffinity column sample preparation using the same antibody used by Klarić, Cvetnić, Pepeljnjak, and Kosalec (2009) and was found to be extremely sensitive at 0.02 μg/L. In order to recover high sensitivity, most researchers concentrated on modifying the normal ELISA protocol. ELISA formats (such as direct, indirect, competitive, and sandwich) are recognized as an excellent and accurate for screening the mycotoxins, but the procedure is somewhat time‐consuming, not ideal for field testing and requires specialist plate readers. Therefore, a transduction system was integrated with appropriate molecular recognition elements (immunochemical) that favored for portable and field analysis. Different analytical methods for OTA, FB_1_, patulin, trichothecenes detection described in (Tables 4, 5, 6).

**Table 4:** Detection of ochratoxin by different analytical methods

Toxin	Matrix	Analytical method	Detection method	Limit of detection	Reference
OTA	Wine	LC	FD	0.07 ng/ml	Aresta, Vatinno, Palmisano, and Zambonin (2006)
OTA	Wheat	HPLC	FLD	23 pg	De Girolamo, McKeague, Miller, DeRosa, and Visconti (2011)
OTA	Model sample	Nanostructured ZnO supporting antibodies	Electrochemical	0.006 ng/ml	Ansari, Kaushik, Solanki, and Malhotra (2010)
OTA	Wines and other foods	HPLC	FD	0.09 μg/L	Tessini et al. (2010)
OTA	Cereal and beverages	Competitive immunoassay linked to gold nanoparticles	SPR	0.042 ng/ml	Yuan, Deng, Lauren, Aguilar, and Wu (2009)
OTA	Green coffee extract	Automated microarray chip reader	Chemiluminescence detection	0.3 μg/L	Sauceda‐Friebe et al. (2011)
OTA	Wine	HPLC	MS/MS	0.005 ng/ml	Campone, Piccinelli, and Rastrelli (2011)

**Table 5:** Detection of fumonisins, patulin by different analytical method

Toxin	Matrix	Analytical method	Detection method	Limit of detection	Reference
FBs	Maize	Direct competitive magneto‐immunoassay	Electrochemical	0.33 µg/L	Wang, Wang et al., (2014); Jodra et al. (2015)
FBs	Maize	Lateral flow immunoassay	Colorimetric	199 µg/kg	Molinelli et al. (2009)
Patulin	Model samples	Competitive immunoassay	SPR	0.1 nM	Pennacchio et al. (2014)
FB_1_	Model samples	Competitive ELISA transferred to microarray	UV absorbance	43 ng/ml	Lamberti et al. (2009)
Patulin	Apple Juice	TLC	CCD	14 µg/L	Welke, Hoeltz, Dottori, and Noll (2009)
Patulin	Apple puree	Photonics immobilization technique	Quartz‐crystal microbalance (QCM)	56 ng/ml	Funari et al. (2015)
FBs	Corn	Indirect competitive ELISA	Electrochemical	5 µg/L	Kadir and Tothill (2010)
FB_1_	Model samples	Immunomagnetic bead based indirect competitive ELISA	Optical immunosensor	0.24 ng/ml	Wang, Liu, et al. (2014)

**Table 6:** Detection of zearalenone and trichothecenes by different analytical methods

Toxin	Matrix	Analytical method	Detection method	Limit of detection	Reference
Trichothecenes	Wheat and maize grains	LC	MS/MS	0.2–3.3 µg/kg	Santini, Ferracane, Somma, Aragón, and Ritieni (2009)
ZEA	Corn	Electro‐polymerization onto surface	SPR	0.3 ng/ml	Chun, Choi, Chang, Choi, and Eremin (2009)
ZEA	Feed	Competitive immunoassay linked to gold nanoparticles	Surface‐enhanced Raman Spectroscopy	1 pg/ml	Liu et al. (2014)
ZEA	Barley, Maize and Wheat Flour	LC	FD	100 µg/kg	Macdonald et al. (2005)
ZEA	Maize	Indirect competitive ELISA	UV absorbance	0.02 µg/L	Tang et al. (2014)
DON	Wheat and maize	Immunochromatographic strip		50 ng/ml	Xu et al. (2010)
DON	Wheat	Direct binding	Electrochemical	6.25 ng/ml	Olcer et al. (2014)

#### 4.2.2 Microplate reader

Microtiter readers can detect the intensity of fluorescence or chemiluminescence and optical absorbance. Microtiter plates possess the unique characteristic of binding proteins evenly (e.g., antibodies or antigens contrary to AFs or secondary antibody). Chemiluminescence immunoassay is another quantitative method depends on ELISA. Mostly, chemiluminescence immunoassay performs better sensitivity than ELISA with a luminol‐based substrate, a secondary antibody (Abs) labeled with horseradish peroxidase, and 384‐well black polystyrene microtiter plates. As chemiluminescence detection does not need either the excitation of fluorescent labels or external light sources, it is more portable and convenient and reduces the complexity of integrated optical components than fluorescence microarrays to certain level (Roda et al., 2006; Yang, Sun, Kostov, & Rasooly, 2011).

#### 4.2.3 Lateral flow strip

Lateral flow strip assay for immunochromatography has fascinated excessive concern in current years. This technique is based on the use of antigen–antibody reactions for the quick analysis of analytes with high sensitivity and specificity. Lateral flow strip assay has developed as the widely and commercially consumed immunoassays for fast analysis of mycotoxins, such as DON (Kolosova, Saeger, Sibanda, Verheijen, & Peteghem, 2007; Kolosova et al., 2008; Xu et al., 2010), ZEA (Kolosova et al., 2007), T‐2 Toxin (Molinelli, Grossalber, & Krska, 2009), and OTA (Cho et al., 2005; Liu, Tsao, Wang, & Yu, 2008). AFB_1_ (Delmulle, De Saeger, Sibanda, Barna‐Vetro, & Van Peteghem, 2005). Lateral flow strip assay has several benefits, such as simple step procedures, manageable setup, and quantity of target analytes can be detected straight with the bare eyes and rapid on‐site detection (5–15 min), low cost and less interference due to chromatography separation.

## 5 NEW DETECTION METHODS FOR MYCOTOXINS QUANTITATIVE ANALYSIS

In the recent years, with the fast advancement of detection technologies and introduction of biotechnology, the detection technology of mycotoxins has grown rapidly. Some of the new technologies that have been applied for the detection of mycotoxins are elaborated in the following sections.

### 5.1 Ultrafast liquid chromatography connected with tandem mass spectrometry (UFLC‐MS/MS)

Ultrafast performance liquid chromatography (UFLC) has made substantial improvements in column technology to accomplish a dramatic increase in speed, resolution, and in separation performance that do not hinge on pressure as in liquid chromatography. UFLC shows the minimum deviation from Van Deemter theory with distinctive and exceptional features thereby shortening the analysis time (Gangadasu, Nagarjuna Reddy, & Dhanalakshmi, 2015). Li, Kong, et al. (2016) has developed an UFLC–MS/MS technique for better productive analysis of OTA, FB_1_ and FB_2_, AFs (AFB_1_, AFB_2_, AFG_1,_ and AFG_2_), and ZEA in yam flours, yam‐derived products, and Chinese yam. Subsequently, optimization of some central factors such as chromatographic separation, MS/MS circumstances, and sample preparation was confirmed to display an outstanding results in means of LOD (≤0.15 ng/ml), linearity (*r* ≥ .9977) and quantification (≤0.5 ng/ml) with better accuracy and precision beside with small run interval (8 min/sample). This reported method was also noted to be proficient for instantaneous detection of mycotoxins another categories of compound matrices. Xing et al. (2016) have reported the quantification of 21 mycotoxins (AFB_1_, AFB_2_, AFG_1_, AFG_2_, T‐2, FB_1_, FB_2_, ST, PA, DAS, NEO, HT‐2, OTA, DON, ZEA, PAT, 3‐ADON, 15‐ ADON, NIV, FUS‐X, CPA) in Radix Paeoniae Alba (RPA) by linking the transformed QuEChERS process with ultrahigh‐performance liquid chromatography–quadrupole‐linear ion trap–mass spectrometry (UHPLC‐QqLIT‐MS) to develop a better yield way for instantaneous detection of mycotoxins. The detection limit and quantification varied from 0.031 to 5.4 μg/kg and 0.20 to 22 μg/kg, respectively. The method proposed herein with noteworthy benefits such as rapid determination, simple pretreatment, and accuracy along with better sensitivity and quantity would be a favored method for the detection and measurement of multiple mycotoxin contaminants in different matrices. The MS/MS method shows numerous mycotoxin detection potentials. The pros and cons of conventional and emerging techniques for mycotoxins analysis are precised in Table 7.

**Table 7:** Pros and cons of conventional and emerging methods for mycotoxins analysis

Conventional/Emerging methods	Pros	Cons
Thin‐layer chromatography (TLC)	Less time consuming, Less equipments required, characteristic fluorescence spot under UV light	Separation takes place up to certain length because of plate length limitation. The separation occurs in an open system or in open condition, and therefore, there is a risk that the humidity and temperature can affect the sample
Liquid chromatography/Mass spectrometry (LC/MS)	Simultaneous analysis of mycotoxins, good sensitivity, provides confirmation, no derivatization required	Very expensive, specialist expertise required, sensitivity relies on ionization technique, matrix‐assisted calibration curve (for quantitative analysis)
High‐performance liquid chromatography (HPLC)	HPLC is extremely fast and efficient compared to other chromatographic techniques, such as TLC. The cycle can be completed in approximately 10–30 min, providing high resolution. It is accurate and easily reproducible. It is largely automated, with minimal training, basic HPLC runs can be performed.	Expensive equipment, HPLC can be costly, requiring large quantities of expensive organics. Troubleshooting problems may be difficult due to the presence of different modules, columns, and mobile phases in the instrument
Gas chromatography–mass spectrometry (GC‐MS)	Simultaneous analysis of mycotoxins, good sensitivity, provides confirmation (MS detector). While GC can separate volatile and nonvolatile components in a sample, MS assists in fragmenting and identifying components based on their mass. It can provide the information about the structure of the compound	Expensive equipment, derivatization required matrix interference problems, nonlinear calibration curve, drifting response, variation in reproducibility and repeatability
Enzyme‐linked immunosorbent assay (ELISA)	Simple sample preparation, inexpensive equipment, high sensitivity, simultaneous analysis of multiple samples, suitable for screening. ELISA has the added advantages of not having to use radioisotopes (radioactive substances) or an expensive radiation counter (radiation counter)	Cross‐reactivity with related mycotoxins, matrix interference problems, possible false‐positive/negative results
Micro plate reader	It improves simple ELISA method by reducing the coating, blocking, and competition time. It can reach a higher sensitivity than ELISA	Not portable and convenient device for field application
Lateral flow strip	One‐step assay, no washing step necessary, fast and low cost, low sample volume, simple test procedure	Qualitative or semi quantitative results, imprecise sample volume reduces precision
Immunosensor	Immunosensors have the following advantages: portability due to their small scale, high selectivity and sensitivity, quick detection, and cheap materials, no cleanup procedure	Cross‐reactivity with related mycotoxins, variation in reproducibility and repeatability, due to small sizes of most the mycotoxin, it is difficult to develop antibody against them; skilled personnel are required to handle the sophisticated equipment
Fluorescence polarization immunoassay	Multi‐analyte immunoassay is feasible, wide detection range, long‐lived luminescence in comparison with conventional fluorophore	Background interference in sample, longer incubation time is required for better reproducibility
Nano particle based methods	The traditional ELISA method is enhanced by gold nanoparticles, Multiple mycotoxins detection using a competitive immunoassay format	Difficult to synthesize and not cost‐effective
Molecular imprinting (MIP)	Cleanup, easy operation, low cost, stable, reusable, high affinity and selectivity toward the target molecule, Polymers are cost‐effective to synthesized and store for several years at room temperature	Poor selectivity, large volume of organic solvents, and long extraction time is required
Microarray technology	High‐throughput screening miniaturized, multiplexed, and parallel processing method	Not common because of their variability and reproducibility, intensive labor requirement

### 5.2 Fluorescence polarization immunoassay

Time‐resolved fluorescence immunoassay **(**TRFIA) is a novel analytical method that has been established since 1980s. This technique uses trivalent rare‐earth metal ions (Eu3+, Tb3+, Sm3+, Dy3+) as tracers. Rare‐earth ions–chelator–antigen chelates are prepared by mixing rare‐earth ions, antigen, and chelator. The tested antigen and labeled antigen compete for the antibody to form immune complexes, and the rare‐earth metal ion presents in the antigen–antibody binding portion of immune complexes is responsible for the fluorescence. Hence, the intensity of fluorescence radiated from the metal ion can be measured by TRFIA (Hagan & Zuchner, 2011). Huang et al. (2009) created TR‐FIA for AFB_1_ and OTA utilizing Eu and Sm as a marker correspondingly. In this reaction, antigen–protein was treated onto micro titer plates followed by the addition of sample and antibody (Monoclonal Abs for OTA and Polyclonal Abs for AFB_1_) after which distinct labeled second antibody was added. This TR‐FIA was verified and displayed a LOD of 0.02 μg/L and 0.05 μg/L for AFB_1_ and OTA, respectively. Multi‐analyte immunoassay is possible using two different markers. This technique provides a wide detection range, good reproducibility, high sensitivity, and prolonged luminescence in comparison with the conventional fluorophore. TRFIA is a quick, simple, economic, and stable technology that can be used to identify mycotoxins in a huge number of samples. Further improvement has been done by researchers to combine immunochemical recognition elements and Raman spectroscopy method. Chauhan et al. (2015) used Raman spectroscopy for AFB_1_ detection. Li, Wen, et al. (2016) has developed multi‐analytes immunoassays and have gained high consideration due to their low sample consumption and short assay times and reduced detection costs per assay. In optimum circumstances, the LOD with MWFPIA was 17.8, 331.5, and 242.0 μg/kg for T‐2 toxin, FB_1,_ and DON, respectively, giving adequate sensitivity for these three contaminants in maize as fixed by the EU. The overall period of analysis and sample preparation was noted to be less than 30 min. Various alterations to homologous MWFPIA such as a way with better sensitivity reduce the detection interval, and targets of interest will result in a simple procedure which can be completed effortlessly.

### 5.3 Nanoparticles based detection methods

Gold nanoparticles were used to enhance the traditional ELISA method. Label‐free sensor has the same sensitivity to a typical merchantable competitive ELISA kit. Another label‐free process was revealed by Xu, Liu, Li, and Ying (2013) who used gold nanorods treated with antibodies to determine AFB_1_. Jodra, López, and Escarpa (2015) used an equivalent technique to encapsulate magnetically labeled elements conveying particular antibody enzyme composite, and electrochemical method was used for AFB_1_ detection.

Surface plasmon resonance (SPR) is an example of an optical detection method that happens when a polarized light hits a prism enclosed by a thin metal (gold) layer. In a few circumstances (polarization, incidence angle, and wavelength), nonbounded electrons at the level of the biochip absorb incident photons and change them into surface plasmon waves. The SPR imaging (SPRi) technique makes SPR method a stage advance. The CCD camera is used to visualize the whole chip and is a sensitive label‐free method. This arrangement permits the biochips to be organized in an array setup in which every active site provides SPR information instantaneously. Detection of multiple toxic mycotoxins is really important to control food quality. A gold nanoparticle (AuNP) intensified SPRi chip was reported to analyze numerous mycotoxins by a competitive immunoassay setup (Hu et al., 2014). Highly sensitive and specific immediate analysis is attained for three characteristic mycotoxins comprising OTA, ZEA, and AFB_1_ with low detection limits of 30, 15, and 8 pg/ml, respectively. SPRi is an innovative device for simultaneous numerous analysis with better accuracies even though problems occur due to minute extents of mycotoxins with single epitope for an unresponsive competitive immunoassay and restricted sensitivity due to the instrumental restraint.

### 5.4 Lateral flow immunochromatographic assay detection method

Lateral flow immunochromatographic assay (LFICA) technique has been extensively used for the analysis of mycotoxins in feeds, foods, and agronomic goods because of its low cost, simplicity, and speed. The modern developments of LFICA with various nanomaterials labeled in the detection of mycotoxins were overviewed and prospected (Xie, Yang, Kong, Yang, & Yang, 2015). Detection sensitivity and specificity were enhanced continuously, and the limit of detection could extend to pictogram (pg) level. LFICA, with the benefits such as rapidness of analysis, high sensitivity, solidity, and low cost, offered a fast detection of mycotoxins for mass screening. Nevertheless, this technique still undergoes certain difficulties, and it requires certain instruments in the field application and in quick and spot analysis of mycotoxins. Magro et al. (2016) have developed “Surface Active Magnetite Nanoparticles” (SAMNs), a consistent and effective mean for citrinin exclusion from *Monascus* preserved foods. The nanomaterial efficacy for citrinin binding was exhibited on (SAMN‐citrinin) complex and *Monascus* suspensions and was characterized by Magnetization measurements and Mossbauer spectroscopy. SAMNs are considered to be an outstanding and steady magnetic nanocarrier for toxin elimination which can be functioned infood industry.

### 5.5 Implementation of microfluidic “lab‐on‐a‐chip” for the detection of mycotoxins in foods

An enormous determination has been dedicated to ultra‐accurate and ultrafast quantitation of trace amount of mycotoxins in foodstuffs, and microfluidic devices have developed as a favorable up‐to‐date analytical platform. The awareness of microfluidic analytical platform develops from the theory of Total Analysis System (TAS), which tends to minimize and assimilate the essential phases for exploration of a sample onto a particular instrument. The microfluidic analytical platform, also recognized as Micro Total Analysis Systems (mTAS), additionally increases its usage thereby creating the entire arrangement of a research laboratory onto a distinct chip in micrometer level (Kovarik et al., 2013; Dittrich, Tachikawa, & Manz, 2006). As its term designates, microfluidics compacts with regulatory solutions of minute quantity (naturally in nanoliters) in micro scale passages (Squires & Quake, 2005). The typical passage dimension of microfluidic analytical instruments ranges from 1 to 1,000 µm (Bayraktar & Pidugu, 2006). Electrochemical‐dependent detection technique is an outstanding method to be merged into the microfluidic LOC instruments because of its inbuilt ability for small scale without affecting performance depletion, better sensitivity and compatibility (Neagu, Perrino, Micheli, Palleschi, & Moscone, 2009; Yeh, Chen, Lin, Chang, & Lin, 2009). It has specific benefits as its reaction is not restricted by sample turbidity or optical path length (Hervás, López, & Escarpa, 2012). MS detection method is noteworthy for its sensitivity and fast speed. The combination of microfabricated devices with this instrument can attain a low limit of detection. MS analysis is a label‐free method joined with microfluidic immunosensor, an alternative mode for the improvement of mycotoxins assays. Liu, Lin, Chan, Lin, and Fuh (2013) explored a chip‐nano liquid chromatography interface/triple quadrupole MS (Chipnano LC/QqQ‐MS) system to regulate AFs in peanut foodstuffs. The application of two column design chip‐based on LC methods decreased matrice interference and online sample preconcentration, thereby enabling concurrent and fast determination of individual AFs. Microfluidic immunosensor joined with chemiluminescence‐based indirect competitive. Molecularly imprinted polymers (MIPs), aptamer, antibody, and microarray technology are four favorable components which can be incorporated with microfluidic LOC instruments to pass the molecule‐specific seizure. Antibodies are the single immunological dependent parting components that are useful into microfluidic devices. The main benefit of antibody based immunoassay is its higher specificity. Addition of dissimilar antibodies into a multi‐channel microfluidic device can increase the quantitative determination of mycotoxins in a better performing way. The classic microfluidic immunosensor applied for the quantification of mycotoxins are lateral flow test strip (LFTS) and capillary electro migration microchip (CE chip) that are forced by capillary force and capillary electro migration respectively (Luppa, Sokoll, & Chan, 2001; Li et al., 2012). For illustration, a competitive immunoassay–microfluidic instruments have been evolved for the segregation and quantification of ZEA (Hervas, Lopez, & Escarpa, 2011). Molecular imprinting (MIP) is an alternative well‐organized separation technique that has been combined into the microfluidic LOC system. MIP is an advanced template induced formation of specific recognition sites where the template guides the alignment and positioning of the substance in structural elements of a self‐build machinery (Ulbricht, Matuschewski, Oechel, & Hicke, 1996). A MIP electrochemical sensor has been recently fabricated for the selective detection of T‐2 toxin by introducing iron ions (Fe^3+^) to enhance the chelation of the templates and metal ions (Gao et al., 2014). Theoretically, the key benefits of MIPs are as follows: it is cost‐effective and the most generic technique for formulating synthetic receptors. MIPs can be prepared for a wide range of compounds with natural biomolecules, but frequently has better specificity in organic solvents and stability in a wide range of pH, temperature, and pressure. However, MIPs also have some limitations like the exact stereochemical structure of the imprint are not known, and template costs may vary considerably from one compound of interest to another, slow mass transfer in the polymer matrice, and unfavorable adsorption isotherm in case of separation media.

Aptamer is a form of single‐stranded oligonucleotides, such as single‐strand deoxyribonucleic acid (ssDNA) and ribonucleic acid (RNA), 25–80 bases long and are manufactured by in vitro selection process known as Systematic Evolution of Ligands by Exponential Enrichment (SELEX). Due to their unique three‐dimensional (3‐D) structures, aptamer can bind target molecules with specificity and high affinity with folding patterns and different spatial structures (Lou et al., 2009). Chapuis‐Hugon, Boisbaudry, Madru, and Pichon (2011) found that aptamer immobilization via noncovalent binding (streptavidin‐activated agarose) has lesser binding efficiency (29%) than covalent binding (cyanogen bromide‐activated sepharose) and less favorable to purify OTA in red wines. De Girolamo et al. (2011) and De Girolamo, Le, Penner, Schena, and Visconti (2012) immobilized aptamer with a coupling gel and used it as a sorbent for the preparation of SPE columns to clean up OTA from wheat sample. Also, LOD of 23 pg/g with average recovery from 74% to 88% was attained by HPLC‐FLD analysis. This column could be reprocessed five times without any damage of enactment. The application of aptamers analysis shows better visions than antibodies in the fields of environmental monitoring, new drug development, food analysis, toxicological study, clinical diagnosis and treatment, etc. Current application of microfluidic‐dependent analytical devices for the determination of mycotoxins has focused the combinative utilization of microfluidic systems with other contemporary techniques known as immunoassays and nanotechnology. The greatest beneficial sorts beyond the customary immunoassays achieved in microwaves are the condensed diffusion distance because of the high surface to volume ratio of the reduced microfluidic systems, which improved analysis time and analytical sensitivity (Hervás et al., 2012).

Microarray technology is a new lab‐on‐chip technology, created on a solid substrate (usually a silicon thin film or glass slide) that assays huge amounts of biological material using parallel and multiplexed processing, high‐throughput screening detection methods. Use of antibody microarrays for the autonomous detection of two common mycotoxins, FB_1_ and AFB_1,_ was reported by Lamberti, Tanzarella, Solinas, Padula, and Mosiello (2009) with LOD of 43 and 3 ng/ml, respectively. The excellence of the microarray information is analogous to data produced using microplate‐based immunoassay (ELISA). Microarray technique has numerous benefits such as the requirement of minimal volume of sample, high‐throughput analysis and possible detection of extensive range of compounds (Nicolaisen, Justesen, Thrane, Skouboe, & Holmstrøm, 2005). However, most of the investigators do not have confidence in the data obtained from microarrays because of their high variability and low reproducibility. Though abovementioned separation procedures have not been extensively applied in the separation and recognition of mycotoxins, these methods are utilized for the separation and enhancement of other nutrient chemical hazards, known as herbicides, antibiotic residues, and pesticides. These components have the prospective to be assimilated into a microfluidic LOC platform for a comprehensive parting of mycotoxins from foodstuffs formerly a critical analysis.

### 5.6 Phage display techniques

Phage display addresses to protein libraries and large peptide on the surface of filamentous phage, which leads to the selection of proteins and peptides, containing antibodies, with high specificity and affinity to practically any target. Hu et al. (2011) created a biological phage display technology (PDT) with a robust separation purpose for peptides. The main aim of this technology is to combine genotype of phage carrier with the phenotype of protein molecule and to shorten the estimation and selection of protein expression library through a series of genetic and biochemical operation. Phage display technology is used to simulate antigen epitope, formulate antibody to skip the cell synthesis and to screen single antibody as target molecule or simulate epitope of mycotoxins using monoclonal antibody (McAb), which can ensure the safety of operators and substitute the standard ingredients of mycotoxins to form nontoxic ELISA detection method. He, Xu, Zhang, Li, and Huang (2014) obtained nine positive clones of ZEA phage from 7‐peptide library after 3‐wheel screening. The results of the competitive ELISA showed that all the nine positive clones could be restrained by ZEA with a detection range of 0.1–10 μg/L. A successful illustration of a simulation epitope of orange penicillin through the PDT provide the basis for developing nontoxic ELISA kit for detecting mycotoxins. Lai, Xu, Xiong, Chen, and Liu (2008) and Lai, Fung, Yang, Renrong, and Xiong (2009) applied the screened OTA simulation epitope peptide for emerging a nontoxic colloidal gold paper slip system. The recently recognized colloidal gold paper slip system could be an appropriate method for the detection of OTA. Using the simulation epitope peptide to substitute the standard toxin product not only reduces environmental pollution of mycotoxins but also recovers the safety of operators. Hence, the combination of PDT with the immune colloidal gold marker technology can result in an imminent drift for detecting mycotoxins.

## 6 CONCLUSION

This review summarizes the recent trends of developments in the methods of sample extraction, cleanup processes, detection technologies, quantitative methods, and also on the current research of fast and noninvasive detection methods. Sample pretreatment has continuously been a challenging step for analysis of mycotoxins in various food matrices. Sample preparation protocol often needs to be optimized to increase the extraction efficiency. Significant advances were made with the introduction of modern IAC and SPE in the cleanup process. Novel nanomaterials have been introduced as absorbents which have been shown to increase specificity in comparison with traditional methods as it can be recycled to reduce the cost. Different analytical approaches for mycotoxins occurring in cereals and cereal‐based products have been advanced and progressively enhanced. Chromatographic methods such as TLC and HPLC are noted to be regular and the global gold techniques in AFs analysis in laboratories. TLC was the most commonly used chromatographic technique applied to mycotoxins in the early 1980s; however, it has certain drawbacks, such as low sensitivity and poor accuracy. TLC method is effectively replaced by HPLC technique. Furthermost often applied detectors are UV, PDA, and FL which ensure a specific use in the area of mycotoxins. The developments have passed from the detection of the single compound determination to the concurrent detection of numerous targets, carried out using immense composite cleanup stages, for example, QuEChERS. Among the ordinary methods, immunoaffinity column cleanup linked with HPLC has been the utmost regularly utilized method for the analysis of major mycotoxins in food and feed. LC‐MS has all the benefits over HPLC for trace level detection and confirmation, particularly for complex matrices, and it can obtain data regarding mycotoxin identity. It has shown great potential for multi‐mycotoxin analysis after single step extraction. A total of 33 mycotoxins including AFs (AFB_1_, AFB_2_, AFG_1,_ AFG_2_), DON, OTA, ZEA, HT‐2, and T‐2 toxin were analyzed and quantified in different food matrices. LC–MS/MS has a great potential to test large amounts of samples for the presence of a variety of mycotoxins. Analytical methods based on spectroscopy and immunochemistry have been added to the earlier chromatographic methods, of which immunoassays emerged as better substitutes for routine and on‐site detection of mycotoxins. Improvement in analytical chemistry and recent advances in immunochemistry have led to more specific, sensitive, simple, and rapid immunoassays that deliver quantitative and semiquantitative results on‐site and have developed as the process of selection for routine analysis of mycotoxins in the field and storehouses. ELISA method has been used for the analysis of AFs (AFB_1_, AFB_2_, AFG_1,_ AFG_2_) and OTA in rice and food stuffs; still, these approaches need confirmatory analysis using other vigorous procedures. It is worth noting that although several sensitive methods like the microplate reader and lateral flow strip have been mentioned in the analysis of mycotoxins based on immunochemical format, they require expertise and well‐instructed operators. Therefore, the quest for label‐free, fast, and more sensitive tools based on immune‐biosensor format continues. Which can offer compact, lightweight, responsive, and reliable mycotoxin detection devices in the field.

Apart from typical antibodies, several new recognition components such as molecularly imprinted polymers and aptamers are applied in mycotoxin detections at pg/ml level. Efforts are continuing on optimizing aptasensors that bind to AFB_1_ for detection in the field. Nanoparticles and nanostructure‐based analytical devices have high sensitivity and low detection limits and can be potentially used as portable instrumentation. The microarray technology is fast, sensitive, but not yet common because of their variability and reproducibility issues.

No doubt, the researchers have shown the pathway of new science, unique technology, inventive material, and state of art sensing technique but are not in reach in real life. Although recommending a single method applicable for all types of samples is not possible, the selection of method should be based on the type of sample, the objective, and the facility available in the laboratory. This critical review will be beneficial for the researchers, and industries involved in mycotoxin research to choose appropriate detection and quantification technique.

## CONFLICT OF INTEREST

The authors declare that they have no conflict of interest.

## ETHICAL APPROVAL

Human and animal testing is unnecessary in our study.

## References

[fsn31474-bib-0002] Abramson, D. (1987). Measurement of ochratoxin A in barley extracts by liquid chromatography‐mass spectrometry. Journal of Chromatography A, 391, 315–320. 10.1016/s0021-9673(01)94330-4 3584327

[fsn31474-bib-0003] Akbas, M. Y. , & Ozdemir, M. (2006). Effect of different ozone treatments on aflatoxin degradation and physicochemical properties of pistachios. Journal of the Science of Food and Agriculture, 86(13), 2099–2104. 10.1002/jsfa.2579

[fsn31474-bib-0004] Alcaide‐Molina, M. , Ruiz‐Jiménez, J. , Mata‐Granados, J. M. , & Luque de Castro, M. D. (2009). High through‐put aflatoxin determination in plant material by automated solid‐phase extraction on‐line coupled to laser‐induced fluorescence screening and determination by liquid chromatography–triple quadrupole mass spectrometry. Journal of Chromatography A, 1216(7), 1115–1125. 10.1016/j.chroma.2008.12.049 19135679

[fsn31474-bib-0005] Ali, N. , Hashim, N. H. , Saad, B. , Safan, K. , Nakajima, M. , & Yoshizawa, T. (2005). Evaluation of a method to determine the natural occurrence of aflatoxins in commercial traditional herbal medicines from Malaysia and Indonesia. Food and Chemical Toxicology, 43(12), 1763–1772. 10.1016/j.fct.2005.05.019 16019122

[fsn31474-bib-0006] Ansari, A. A. , Kaushik, A. , Solanki, P. R. , & Malhotra, B. D. (2010). Nanostructured zinc oxide platform for mycotoxin detection. Bioelectrochemistry, 77(2), 75–81. 10.1016/j.bioelechem.2009.06.014 19648064

[fsn31474-bib-0007] Aresta, A. , Vatinno, R. , Palmisano, F. , & Zambonin, C. G. (2006). Determination of Ochratoxin A in wine at sub ng/mL levels by solid‐phase microextraction coupled to liquid chromatography with fluorescence detection. Journal of Chromatography A, 1115(1–2), 196–201. 10.1016/j.chroma.2006.02.092 16554057

[fsn31474-bib-0008] Arroyo‐Manzanares, N. , Gamiz‐Gracia, L. , & Garcia‐Campana, A. M. (2012). Determination of ochratoxin A in wines by capillary liquid chromatography with laser induced fluorescence detection using dispersive liquid‐liquid microextraction. Food Chemistry, 135(2), 368–372. 10.1016/j.foodchem.2012.05.009 22868101

[fsn31474-bib-0009] Azaiez, I. , Giusti, F. , Sagratini, G. , Mañes, J. , & Fernández‐Franzón, M. (2014). Multi‐mycotoxins analysis in dried fruit by LC/MS/MS and a modified QuEChERS procedure. Food Analytical Methods, 7(4), 935–945. 10.1007/s12161-013-9785-3

[fsn31474-bib-0010] Battilani, P. , Gualla, A. , Dall'Asta, C. , Pellacani, C. , Galaverna, G. , Giorni, P. , … Costa, L. (2011). Phomopsins: An overview of phytopathological and chemical aspects, toxicity, analysis and occurrence. World Mycotoxin Journal, 4(4), 345–359. 10.3920/WMJ2011.1302.

[fsn31474-bib-0011] Bayraktar, T. , & Pidugu, S. B. (2006). Characterization of liquid flows in microfluidic systems. International Journal of Heat and Mass Transfer, 49(5–6), 815–824. 10.1016/j.ijheatmasstransfer.2005.11.007

[fsn31474-bib-0012] Bennett, J. , & Klich, M. (2003). Mycotoxins. Clinical Microbiology Reviews, 16, 497–516. 10.1128/CMR.16.3.497-516.2003 12857779 PMC164220

[fsn31474-bib-0013] Berthiller, F. , Schuhmacher, R. , Buttinger, G. , & Krska, R. (2005). Rapid simultaneous determination of major type A‐ and B‐trichothecenes as well as zearalenone in maize by high performance liquid chromatography‐tandem mass spectrometry. Journal of Chromatography A, 1062(2), 209–216. 10.1016/j.chroma.2004.11.011 15679158

[fsn31474-bib-0014] Bhat, R. , Rai, R. V. ,& Karim, A. A. (2010). Mycotoxins in food and feed: Present status and future concerns. Comprehensive Reviews in Food Science and Food Safety, 9(1), 57–81. 10.1111/j.1541-4337.2009.00094 33467806

[fsn31474-bib-0015] Bozza, A. , Tralamazza, S. M. , Rodriguez, J. I. , Scholz, M. B. S. , Reynaud, D. T. , Dalzoto, P. R. , & Pimentel, I. C. (2013). Potential of Fourier transform infrared spectroscopy (FTIR) to detection and quantification of ochratoxin A: A comparison between reflectance and transmittance techniques. International Journal of Pharmaceutical, Chemical & Biological Sciences, 3(4), 1242–1247.

[fsn31474-bib-0016] Bredenkamp, M. W. , Dillen, J. L. M. , van Rooyen, P. H. , & Steyn, P. S. (1989). Crystal structures and conformational analysis of ochratoxin A and B: Probing the chemical structure causing toxicity. Journal of the Chemical Society, 2(11), 1835–1839. 10.1039/p29890001835

[fsn31474-bib-0017] Brera, C. , Debegnach, F. , Minardi, V. , Pannunzi, E. , De Santis, B. , Miraglia, M. , & Zanon, F. (2007). Immunoaffinity column cleanup with liquid chromatography for determination of aflatoxin B1 in corn samples: Interlaboratory study. Journal of AOAC International, 90(3), 765–772. 17580628

[fsn31474-bib-0018] Budavari, S. (1989). The Merck index In An Encyclopedia of chemicals, drugs and biologicals. 11th edn (pp. 2–3). Rahway, NJ: Merck and Co. Inc. ISBN 911910-28-X, 2274

[fsn31474-bib-0019] Campone, L. , Piccinelli, A. L. , & Rastrelli, L. (2011). Dispersive liquid–liquid microextraction combined with high‐performance liquid chromatography–tandem mass spectrometry for the identification and the accurate quantification by isotope dilution assay of Ochratoxin A in wine samples. Analytical and Bioanalytical Chemistry, 399, 1279–1286. 10.1007/s00216-010-4347-7 21052643

[fsn31474-bib-0020] Caputo, D. , de Cesare, G. , Nascetti, A. , Scipinotti, R. , Pavanello, F. , & Arrigoni, R. (2014). DEMOCHEM: Integrated system for mycotoxins detection. Procedia Engineering, 87, 1354–1357. 10.1016/j.proeng.2014.11.719

[fsn31474-bib-0021] Carlton, W. W. , & Tuite, J. (1977). Metabolites of *P. viridicatum* toxicology In Rodricks J.V., Hesseltine C. W., & Mehlman M.A., (Eds.), Mycotoxins in human and animal health (pp. 525–555). Park Forest South, IL: Pathotox Publications Inc.

[fsn31474-bib-0022] Chapuis‐Hugon, F. , du Boisbaudry, A. , Madru, B. , & Pichon, V. (2011). New extraction sorbent based on aptamers for the determination of ochratoxin A in red wine. Analytical and Bioanalytical Chemistry, 400(5), 1199–1207. 10.1007/s00216-010-4574-y 21221554

[fsn31474-bib-0023] Chauhan, R. , Solanki, P. R. , Singh, J. , Mukherjee, I. , Basu, T. , & Malhotra, B. D. (2015). A novel electrochemical piezoelectric label free immunosensor for aflatoxin B1 detection in groundnut. Food Control, 52, 60–70. 10.1016/j.foodcont.2014.12.009

[fsn31474-bib-0024] Cho, Y. J. , Lee, D. H. , Kim, D. O. , Min, W. K. , Bong, K. T. , Lee, G. G. , & Seo, J. H. (2005). Production of a monoclonal antibody against ochratoxin A and its application to immunochromatographic assay. Journal of Agricultural and Food Chemistry, 53(22), 8447–8451. 10.1021/jf051681q 16248536

[fsn31474-bib-0025] Chun, H. S. , Choi, E. H. , Chang, H. J. , Choi, S. W. , & Eremin, S. A. (2009). A fluorescence polarization immunoassay for the detection of zearalenone in corn. Analytica Chimica Acta, 639(1–2), 83–89. 10.1016/j.aca.2009.02.048 19345763

[fsn31474-bib-0026] Cirlini, M. , Dall'Asta, C. , & Galaverna, G. (2012). Hyphenated chromatographic techniques for structural characterization and determination of masked mycotoxins. Journal of Chromatography A, 1255, 145–152. 10.1016/j.chroma.2012.02.057 22424768

[fsn31474-bib-0027] Couderc, F. , Caussé, E. , & Bayle, C. (1998). Drug analysis by capillary electrophoresis and laser‐induced fluorescence. Electrophoresis, 19(16–17), 2777–2790. 10.1002/elps.1150191605 9870374

[fsn31474-bib-0028] Creppy, E. E. (1999). Human ochratoxicosis. Journal of Toxicology: Toxin Reviews, 18, 277–293.

[fsn31474-bib-0029] Cucci, C. , Mignani, A. G. , Dall'Asta, C. , Pela, R. , & Dossena, A. (2007). A portable fluorometer for the rapid screening of M1 aflatoxin. Sensors and Actuators B: Chemical, 126(2), 467–472. 10.1016/j.snb.2007.03.036

[fsn31474-bib-0030] Dall'Asta, C. , De Dea Lindner, J. , Galaverna, G. , Dossena, A. , Neviani, E. , & Marchelli, R. (2008). The occurrence of ochratoxin A in blue cheese. Food Chemistry, 106(2), 729–734. 10.1016/j.foodchem.2007.06.049

[fsn31474-bib-0031] De Girolamo, A. D. , Le, L. , Penner, G. , Schena, R. , & Visconti, A. (2012). Analytical performances of a DNA‐ligand system using time‐resolved fluorescence for the determination of ochratoxin A in wheat. Analytical and Bioanalytical Chemistry, 403(9), 2627–2634. 10.1007/s00216-012-6076-6 22576657

[fsn31474-bib-0032] De Girolamo, A. , McKeague, M. , Miller, J. D. , DeRosa, M. C. , & Visconti, A. (2011). Determination of ochratoxin A in wheat after clean‐up through a DNA aptamer‐based solid phase extraction column. Food Chemistry, 127(3), 1378–1384. 10.1016/j.foodchem.2011.01.107 25214141

[fsn31474-bib-0033] De Jesus, A. E. , Steyn, P. S. , Vleggaar, R. , & Wessels, P. L. (1980). Carbon‐13 nuclear magnetic resonance assignments and biosynthesis of the mycotoxin ochratoxin A. Journal of the Chemical Society, Perkin Transactions, 1, 52 10.1039/p19800000052 945292

[fsn31474-bib-0034] De Santis, B. , Debegnach, F. , Gregori, E. , Russo, S. , Marchegiani, F. , Moracci, G. , & Brera, C. (2017). Development of a LC‐MS/MS method for the multi‐mycotoxin determination in composite cereal‐based samples. Toxins, 9(5), 169. 10.3390/toxins9050169 PMC5450717 28524101

[fsn31474-bib-0035] Delmulle, B. S. , De Saeger, S. M. D. G. , Sibanda, L. , Barna‐Vetro, I. , & Van Peteghem, C. H. (2005). Development of an immunoassay‐based lateral flow dipstick for the rapid detection of aflatoxin B1 in pig feed. Journal of Agricultural and Food Chemistry, 53(9), 3364–3368. 10.1021/jf0404804 15853373

[fsn31474-bib-0036] Dittrich, P. S. , Tachikawa, K. , & Manz, A. (2006). Micro total analysis systems. Latest advancements and trends. Analytical Chemistry, 78(12), 3887–3908. 10.1021/AC0605602 16771530

[fsn31474-bib-0037] Dong, F. , Wang, S. , Yu, M. , Sun, Y. , Xu, J. , & Shi, J. (2017). Natural occurrence of deoxynivalenol and deoxynivalenol‐3‐glucoside in various wheat cultivars grown in Jiangsu province, China. World Mycotoxin Journal, 10(3), 285–293. 10.3920/WMJ2016.2158

[fsn31474-bib-0038] Dong, M. , Si, W. , Jiang, K. , Nie, D. , Wu, Y. , Zhao, Z. , … Han, Z. (2015). Multi‐walled carbon nanotubes as solid‐phase extraction sorbents for simultaneous determination of type A trichothecenes in maize, wheat and rice by ultra‐high performance liquid chromatography‐tandem mass spectrometry. Journal of Chromatography A, 1423, 177–182. 10.1016/j.chroma.2015.10.068 26549860

[fsn31474-bib-0039] Edinboro, L. E. , & Karnes, H. T. (2005). Determination of aflatoxin B1 in sidestream cigarette smoke by immunoaffinity column extraction coupled with liquid chromatography/mass spectrometry. Journal of Chromatography A, 1083(1–2), 127–132. 10.1016/j.chroma.2005.06.032 16078698

[fsn31474-bib-0040] Etzel, R. A. , Montana, E. , Sorenson, W. G. , Kullman, G. J. , Allan, T. M. , & Dearborn, D. G. (1998). Acute pulmonary hemorrhage in infants associated with exposure to Stachybotrys atra and other fungi. Archives of Pediatrics & Adolescent Medicine, 152(8), 757–762. 10.1001/archpedi.152.8.757 9701134

[fsn31474-bib-0041] European Commission (EC) (2010). Commission regulation (EU) No 165/2010 of 26 February 2010 amending Regulation (EC) No 1881/2006 setting maximum levels for certain contamination in foodstuffs as regards aflatoxin. Official Journal of the European Union L, 50, 8–12.

[fsn31474-bib-0042] Food and Drug Administration (FDA) (2002). Inventory of effective food contact substance (FCS). Notification No. 178. Retrieved from http://www.accessdata.fda.gov/scripts/fcn/fcnDetailNavigation.cfm?rpt=fcsListing%26xml:id=178

[fsn31474-bib-0043] Funari, R. , Della Ventura, B. , Carrieri, R. , Morra, L. , Lahoz, E. , Gesuele, F. , & Velotta, R. (2015). Detection of parathion and patulin by quartz-crystal microbalance functionalized by the photonics immobilization technique. Biosensors and Bioelectronics, 67, 224–229. 25190088 10.1016/j.bios.2014.08.020

[fsn31474-bib-0044] Gangadasu, B. R. , Nagarjuna Reddy, G. , & Dhanalakshmi, K. (2015). Comparison of UPLC with UFLC: Liquid Chromatography. International Journal of Pharmaceutical Science Review and Research, 31(1), 97–101.

[fsn31474-bib-0045] Gao, X. , Cao, W. , Chen, M. , Xiong, H. , Zhang, X. , & Wang, S. (2014). A high sensitivity electrochemical sensor based on Fe ^3+^ ‐Ion molecularly imprinted film for the detection of T‐2 toxin. Electroanalysis, 26(12), 2739–2746. 10.1002/elan.201400237

[fsn31474-bib-0046] Generotti, S. , Cirlini, M. , Malachova, A. , Sulyok, M. , Berthiller, F. , Dall'Asta, C. , & Suman, M. (2015). Deoxynivalenol & deoxynivalenol‐3‐glucoside mitigation through bakery production strategies: Effective experimental design within industrial rusk‐making technology. Toxins, 7(8), 2773–2790. 26213969 10.3390/toxins7082773 PMC4549723

[fsn31474-bib-0047] Gilbert, J. , & Vargas, E. A. (2003). Advances in sampling and analysis for aflatoxins in food and animal feed. Journal of Toxicology: Toxin Reviews, 22(2–3), 381–422. 10.1081/TXR-120024099

[fsn31474-bib-0048] Göbel, R. , & Lusky, K. (2004). Simultaneous determination of aflatoxins, ochratoxin A, and zearalenone in grains by new immunoaffinity column/liquid chromatography. Journal of AOAC International, 87(2), 411–416. 15164835

[fsn31474-bib-0049] Hagan, A. K. , & Zuchner, T. (2011). Lanthanide‐based time‐resolved luminescence immunoassays. Analytical and Bioanalytical Chemistry, 400(9), 2847–2864. 10.1007/s00216-011-5047-7. 21556751 PMC3102841

[fsn31474-bib-0050] Hagler, J. R. W. M. , Towers, N. R. , Mirocha, C. J. , Eppley, R. M. , & Bryden, W. L. (2001). Zearalenone: Mycotoxin or mycoestrogen? In Summerell B. A., Leslie J. F., Backhouse D., Bryden W. L., & Burgess L. W. (Eds.), Fusarium‐Paul E. Nelson Memorial Symposium. St. Paul, MN: AS Press, The American Phytopathological Society.

[fsn31474-bib-0052] He, Q.‐H. , Xu, Y. , Zhang, C.‐Z. , Li, Y.‐P. , & Huang, Z.‐B. (2014). Phage‐borne peptidomimetics as immunochemical reagent in dot‐immunoassay for mycotoxin zearalenone. Food Control, 39(1), 56–61. 10.1016/j.foodcont.2013.10.019

[fsn31474-bib-0053] Hervas, M. , Lopez, M. A. , & Escarpa, A. (2011). Integrated electrokinetic magnetic bead‐based electrochemical immunoassay on microfluidic chips for reliable control of permitted levels of zearalenone in infant foods. Analyst, 136(10), 2131–2138. 10.1039/c1an15081b 21394379

[fsn31474-bib-0054] Hervás, M. , López, M. A. , & Escarpa, A. (2012). Electrochemical immunosensing on board microfluidic chip platforms. TrAC Trends in Analytical Chemistry, 31, 109–128. 10.1016/j.trac.2011.06.020

[fsn31474-bib-0055] Herzallah, S. M. (2009). Determination of aflatoxins in eggs, milk, meat and meat products using HPLC fluorescent and UV detectors. Food Chemistry, 114(3), 1141–1146. 10.1016/j.foodchem.2008.10.077

[fsn31474-bib-0056] Hocking, A. D. , Holds, K. , & Tobin, N. (1988). Intoxication by tremorgenic mycotoxin (penitrem A) in a dog. Australian Veterinary Journal, 65(3), 82–85. 10.1111/j.1751-0813.1988.tb07366.x 3401148

[fsn31474-bib-0057] Holzapfel, C. W. , Purchase, I. F. H. , Steyn, P. S. , & Gouws, L. (1966). The toxicity and chemical assay of sterigmatocystin, a carcinogenic mycotoxin, and its isolation from two new fungal sources. South African Medical Journal, 40(12), 1100–1101. 6006093

[fsn31474-bib-0058] Hu, W. , Chen, H. , Zhang, H. , He, G. , Li, X. , Zhang, X. , … Li, C. M. (2014). Sensitive detection of multiple mycotoxins by SPRi with gold nanoparticles as signal amplification tags. Journal of Colloid and Interface Science, 431, 71–76. 10.1016/j.jcis.2014.06.007 24992296

[fsn31474-bib-0059] Hu, X. F. , Li, L. , Xing, G. X. , Pei, Y. F. , Zhi, A. M. , Wang, Y. , & Deng, R. G. (2011). The usage of phage display technique in mycotoxins detection. Journal of Henan Agricultural Science, 40(6), 32–35.

[fsn31474-bib-0060] Huang, B. , Xiao, H. , Zhang, J. , Zhang, L. , Yang, H. , Zhang, Y. , & Jin, J. (2009). Dual‐label time‐resolved fluoroimmunoassay for simultaneous detection of aflatoxin B1 and ochratoxin A. Archives of Toxicology, 83(6), 619–624. 10.1007/s00204-009-0410-6 19252900

[fsn31474-bib-0061] Iha, M. H. , Barbosa, C. B. , Heck, A. R. , & Trucksess, M. W. (2014). Aflatoxin M1 and ochratoxin A in human milk in Ribeirão Preto‐SP, Brazil. Food Control, 40, 310–313. 10.1016/j.foodcont.2013.12.014

[fsn31474-bib-0062] Iha, M. H. , Barbosa, C. B. , Okada, I. A. , & Trucksess, M. W. (2011). Occurrence of aflatoxin M1 in dairy products in Brazil. Food Control, 22(12), 1971–1974. 10.1016/j.foodcont.2011.05.013

[fsn31474-bib-0063] Ji, X. , Xu, J. , Wang, X. , Qi, P. , Wei, W. , Chen, X. , … Zhou, Y. U. (2015). Citrinin determination in red fermented rice products by optimized extraction method coupled to liquid chromatography tandem mass spectrometry (LC‐MS/MS). Journal of Food Science, 80(6), T1438–T1444. 10.1111/1750-3841.12900 25943499

[fsn31474-bib-0064] Jiang, W. X. , Wang, Z. H. , Nolke, G. , Zhang, J. , Niu, L. L. , & Shen, J. Z. (2013). Simultaneous determination of aflatoxin B1 and aflatoxin M1 in food matrices by enzyme‐linked immunosorbent assay. Food Analytical Methods, 6(3), 767–774. 10.1007/s12161-012-9484-5

[fsn31474-bib-0065] Jodra, A. , López, M. Á. , & Escarpa, A. (2015). Disposable and reliable electrochemical magnetoimmunosensor for Fumonisins simplified determination in maize‐based foodstuffs. Biosensors and Bioelectronics, 64, 633–638. 10.1016/j.bios.2014.09.054 25441412

[fsn31474-bib-0066] Johny, A. , Fæste, C. K. , Bogevik, A. S. , Berge, G. M. , Fernandes, J. M. , & Ivanova, L. (2019). Development and validation of a liquid chromatography high‐resolution mass spectrometry method for the simultaneous determination of mycotoxins and phytoestrogens in plant‐based fish feed and exposed fish. Toxins, 11(4), 222 10.3390/toxins11040222 PMC6520669 31013949

[fsn31474-bib-0067] Kadir, M. K. , & Tothill, I. E. (2010). Development of an electrochemical immunosensor for fumonisins detection in foods. Toxins, 2(4), 382–398. 22069591 10.3390/toxins2040382 PMC3153203

[fsn31474-bib-0068] Khalesi, M. , Sheikh‐Zeinoddin, M. , & Tabrizchi, M. (2011). Determination of ochratoxin A in licorice root using inverse ion mobility spectrometry. Talanta, 83(3), 988–993. 10.1016/j.talanta.2010.11.004 21147348

[fsn31474-bib-0069] Klarić, M. , Cvetnić, Z. , Pepeljnjak, S. , & Kosalec, I. (2009). Co‐occurrence of aflatoxins, ochratoxin A, fumonisins, and zearalenone in cereals and feed, determined by competitive direct enzyme‐linked immunosorbent assay and thin‐layer chromatography. Archives of Industrial Hygiene and Toxicology, 60(4), 427–434. 10.2478/10004-1254-60-2009-1975 20061243

[fsn31474-bib-0070] Ko, J. , Lee, C. , & Choo, J. (2015). Highly sensitive SERS‐based immunoassay of aflatoxin B1 using silica‐encapsulated hollow gold nanoparticles. Journal of Hazardous Materials, 285, 11–17. 10.1016/j.jhazmat.2014.11.018 25462866

[fsn31474-bib-0071] Kolosova, A. Y. , De Saeger, S. , Sibanda, L. , Verheijen, R. , & Van Peteghem, C. (2007). Development of a colloidal gold‐based lateral‐flow immunoassay for the rapid simultaneous detection of zearalenone and deoxynivalenol. Analytical and Bioanalytical Chemistry, 389(7–8), 2103–2107. 10.1007/s00216-007-1642-z 17922115

[fsn31474-bib-0072] Kolosova, A. Y. , Sibanda, L. , Dumoulin, F. , Lewis, J. , Duveiller, E. , Van Peteghem, C. , & De Saeger, S. (2008). Lateral‐flow colloidal gold‐based immunoassay for the rapid detection of deoxynivalenol with two indicator ranges. Analytica Chimica Acta, 616(2), 235–244. 10.1016/j.aca.2008.04.029 18482609

[fsn31474-bib-0073] Kong, W. J. , Li, J. Y. , Qiu, F. , Wei, J. H. , Xiao, X. H. , Zheng, Y. , & Yang, M. H. (2013). Development of a sensitive and reliable high performance liquid chromatography method with fluorescence detection for high‐throughput analysis of multi‐class mycotoxins in Coix seed. Analytica Chimica Acta, 799, 68–76. 10.1016/j.aca.2013.08.042 24091376

[fsn31474-bib-0074] Kovarik, M. L. , Ornoff, D. M. , Melvin, A. T. , Dobes, N. C. , Wang, Y. , Dickinson, A. J. , … Allbritton, N. L. (2013). Micro total analysis systems: Fundamental advances and applications in the laboratory, clinic, and field. Analytical Chemistry, 85(2), 451–472. 10.1021/ac3031543 23140554 PMC3546124

[fsn31474-bib-0075] Lai, W. , Fung, D. Y. , Yang, X. , Renrong, L. , & Xiong, Y. (2009). Development of a colloidal gold strip for rapid detection of ochratoxin A with mimotope peptide. Food Control, 20(9), 791–795. 10.1016/j.foodcont.2008.10.007

[fsn31474-bib-0076] Lai, W. H. , Xu, Y. , Xiong, Y. H. , Chen, Y. , & Liu, W. J. (2008). Development of colloidal gold strip with mimotope pepfide for rapid detection of ochratoxin A and comparison between it and traditional strip. Food Science, 9, 465–468.

[fsn31474-bib-0077] Lalljie, S. P. D. , & Sandra, P. (1995). Practical and quantitative aspects in the analysis of FITC and DTAF amino acid derivatives by capillary electrophoresis and LIF detection. Chromatographia, 40(9–10), 519–526. 10.1007/BF02290262

[fsn31474-bib-0078] Lamberti, I. , Tanzarella, C. , Solinas, I. , Padula, C. , & Mosiello, L. (2009). An antibody‐based microarray assay for the simultaneous detection of aflatoxin B1 and fumonisin B1. Mycotoxin Research, 25(4), 193–200. 10.1007/s12550-009-0028-9 23605148

[fsn31474-bib-0079] Lattanzio, V. M. T. , Solfrizzo, M. , & Visconti, A. (2008). Determination of trichothecenes in cereals and cereal‐based products by liquid chromatography‐tandem mass spectrometry. Food Additives & Contaminants. Part A, Chemistry, Analysis, Control, Exposure & Risk Assessment, 25(3), 320–330. 10.1080/02652030701513792 17906997

[fsn31474-bib-0080] Lehotay, S. J. , Tully, J. , Garca, A. V. , Contreras, M. , Mol, H. , Heinke, V. , & Poulsen, M. E. (2007). Determination of pesticide residues in foods by acetonitrile extraction and partitioning with magnesium sulfate: Collaborative study. Journal of AOAC International, 90(2), 485–520. 17474521

[fsn31474-bib-0081] Lerda, D. (2011). Mycotoxins factsheet (4th ed.). JRC Technical Notes. Retrieved from http://irmm.jrc.ec.europa.eu/

[fsn31474-bib-0082] Levasseur‐Garcia, C. (2018). Updated overview of infrared spectroscopy methods for detecting mycotoxins on cereals (corn, wheat, and barley). Toxins, 10(1), 38 10.3390/toxins10010038 PMC5793125 29320435

[fsn31474-bib-0083] Li, C. , Wen, K. , Mi, T. , Zhang, X. , Zhang, H. , Zhang, S. , … Wang, Z. (2016). A universal multi‐wavelength fluorescence polarization immunoassay for multiplexed detection of mycotoxins in maize. Biosensors and Bioelectronics, 79, 258–265. 10.1016/j.bios.2015.12.033 26720917

[fsn31474-bib-0084] Li, M. , Kong, W. , Li, Y. , Liu, H. , Liu, Q. , Dou, X. , … Yang, M. (2016). High‐throughput determination of multi‐mycotoxins in Chinese yam and related products by ultra fast liquid chromatography coupled with tandem mass spectrometry after one‐step extraction. Journal of Chromatography B: Analytical Technologies in the Biomedical and Life Sciences, 1022, 118–125. 10.1016/j.jchromb.2016.04.014 27085799

[fsn31474-bib-0085] Li, Y.‐S. , Zhou, Y. U. , Lu, S.‐Y. , Guo, D.‐J. , Ren, H.‐L. , Meng, X.‐M. , … Liu, Z.‐S. (2012). Development of a one‐step test strip for rapid screening of fumonisins B1, B2 and B3 in maize. Food Control, 24(1–2), 72–77.

[fsn31474-bib-0086] Liu, B. H. , Tsao, Z. J. , Wang, J. J. , & Yu, F. Y. (2008). Development of a monoclonal antibody against ochratoxin A and its application in enzyme‐linked immunosorbent assay and gold nanoparticle immunochromatographic strip. Analytical Chemistry, 80(18), 7029–7035. 10.1021/ac800951p 18698802

[fsn31474-bib-0087] Liu, H. Y. , Lin, S. L. , Chan, S. A. , Lin, T. Y. , & Fuh, M. R. (2013). Microfluidic chip‐based nano‐liquid chromatography tandem mass spectrometry for quantification of aflatoxins in peanut products. Talanta, 113, 76–81. 10.1016/j.talanta.2013.03.053 23708626

[fsn31474-bib-0088] Liu, J. , Hu, Y. , Zhu, G. , Zhou, X. , Jia, L. , & Zhang, T. (2014). Highly sensitive detection of zearalenone in feed samples using competitive surface‐enhanced Raman scattering immunoassay. Journal of Agricultural and Food Chemistry, 62(33), 8325–8332. 10.1021/jf503191e 25052032

[fsn31474-bib-0089] Lou, X. , Qian, J. , Xiao, Y. , Viel, L. , Gerdon, A. E. , Lagally, E. T. , … Soh, H. T. (2009). Micromagnetic selection of aptamers in microfluidic channels. Proceedings of the National Academy of Sciences of the United States of America, 106(9), 2989–2994. 10.1073/pnas.0813135106 19202068 PMC2637280

[fsn31474-bib-0090] Luppa, P. B. , Sokoll, L. J. , & Chan, D. W. (2001). Immunosensors—principles and applications to clinical chemistry. Clinica Chimica Acta, 314(1–2), 1–26. 10.1016/S0009-8981(01)00629-5 11718675

[fsn31474-bib-0091] Ma, F. , Chen, R. , Li, P. , Zhang, Q. , Zhang, W. , & Hu, X. (2013). Preparation of an immunoaffinity column with amino‐silica gel microparticles and its application in sample cleanup for aflatoxin detection in agri‐products. Molecules, 18(2), 2222–2235. 10.3390/molecules18022222 23434872 PMC6270504

[fsn31474-bib-0092] Macdonald, S. J. , Anderson, S. , Brereton, P. , Wood, R. , Damant, A. , Aletrari, M. , & Welsh, P. (2005). Determination of zearalenone in barley, maize and wheat flour, polenta, and maize‐based baby food by immunoaffinity column cleanup with liquid chromatography: Interlaboratory study. Journal of AOAC International, 88(6), 1733–1740. 10.1021/jf0479315 16526456

[fsn31474-bib-0093] Magro, M. , Moritz, D. E. , Bonaiuto, E. , Baratella, D. , Terzo, M. , Jakubec, P. , … Vianello, F. (2016). Citrinin mycotoxin recognition and removal by naked magnetic nanoparticles. Food Chemistry, 203, 505–512. 10.1016/j.foodchem.2016.01.147 26948644

[fsn31474-bib-0094] Martin, A. , Aranda, E. , Benito, M. J. , Perez‐Nevado, F. , & Cordoba, M. G. (2005). Identification of fungal contamination and determination of mycotoxigenic molds by micellar electrokinetic capillary chromatography in smoked paprika. Journal of Food Protection, 68(4), 815–822. 10.4315/0362-028X-68.4.815 15830676

[fsn31474-bib-0095] Miller, J. D. (1992). Fungi as contaminants in indoor air. Atmospheric Environment. Part A. General Topics, 26(12), 2163–2172. 10.1016/0960-1686(92)90404-9

[fsn31474-bib-0096] Mishra, H. N. , & Das, C. (2003). A Review on Biological Control and Metabolism of Aflatoxin. Critical Reviews in Food Science and Nutrition, 43(3), 245–264. 10.1080/10408690390826518 12822672

[fsn31474-bib-0097] Mochamad, L. , & Hermanto, B. (2017). High‐performance liquid chromatography ultraviolet‐photodiode array detection method for aflatoxin B1 in cattle feed supplements. Veterinary World, 10(8), 932 10.14202/vetworld.2017.932-938 28919686 PMC5591482

[fsn31474-bib-0098] Molinelli, A. , Grossalber, K. , & Krska, R. (2009). A rapid lateral flow test for the determination of total type B fumonisins in maize. Analytical and Bioanalytical Chemistry, 395(5), 1309–1316. 10.1007/s00216-009-3082-4 19756539

[fsn31474-bib-0100] Munday, K. (1985). Studies on the mechanism of toxicity of the mycotoxin sporidesmin. IV. Inhibition by copper‐chelating agents of the generation of superoxide radical by sporidesmin. Journal of Applied Toxicology, 5(2), 69–73. 10.1002/jat.2550050206 2987332

[fsn31474-bib-0101] Ndube, N. , van der Westhuizen, L. , & Shephard, G. S. (2009). Determination of fumonisins in maize by HPLC with ultraviolet detection of o-phthaldialdehyde derivatives. Mycotoxin research, 25(4), 225. 23605151 10.1007/s12550-009-0031-1

[fsn31474-bib-0102] Neagu, D. , Perrino, S. , Micheli, L. , Palleschi, G. , & Moscone, D. (2009). Aflatoxin M1 determination and stability study in milk samples using a screen‐printed 96‐well electrochemical microplate. International Dairy Journal, 19(12), 753–758. 10.1016/j.idairyj.2009.06.004

[fsn31474-bib-0103] Nicolaisen, M. , Justesen, A. F. , Thrane, U. , Skouboe, P. , & Holmstrøm, K. (2005). An oligonucleotide microarray for the identification and differentiation of trichothecene producing and non‐producing *Fusarium* species occurring on cereal grain. Journal of Microbiological Methods, 62(1), 57–69. 10.1016/j.mimet.2005.01.009 15823394

[fsn31474-bib-0104] Nieto, C. H. D. , Granero, A. M. , Zon, M. A. , & Fernández, H. (2018). Sterigmatocystin: A mycotoxin to be seriously considered. Food and Chemical Toxicology, 118, 460–470. 10.1016/j.fct.2018.05.057 29842907

[fsn31474-bib-0105] Núñez, O. , Gallart‐Ayala, H. , Martins, C. P. , & Lucci, P. (2012). New trends in fast liquid chromatography for food and environmental analysis. Journal of Chromatography A, 1228, 298–323. 10.1016/j.chroma.2011.10.091 22153282

[fsn31474-bib-0106] Olcer, Z. , Esen, E. , Muhammad, T. , Ersoy, A. , Budak, S. , & Uludag, Y. (2014). Fast and sensitive detection of mycotoxins in wheat using microfluidics based real‐time electrochemical profiling. Biosensors and Bioelectronics, 62, 163–169. 10.1016/j.bios.2014.06.025 24998314

[fsn31474-bib-0107] O'Riordan, M. J. , & Wilkinson, M. G. (2009). Comparison of analytical methods for aflatoxin determination in commercial chilli spice preparations and subsequent development of an improved method. Food Control, 20(8), 700–705.

[fsn31474-bib-0108] Pallarés, N. , Font, G. , Mañes, J. , & Ferrer, E. (2017). Multimycotoxin LC–MS/MS analysis in tea beverages after dispersive liquid–liquid Microextraction (DLLME). Journal of Agricultural and Food Chemistry, 65(47), 10282–10289. 10.1021/acs.jafc.7b03507 29068686

[fsn31474-bib-0109] Pascale, M. N. (2009). Detection methods for mycotoxins in cereal grains and cereal products. Zbornik Matice srpske za prirodne nauke, 117, 15–25. 10.2298/ZMSPN0917015P

[fsn31474-bib-0110] Pascale, M. , Haidukowski, M. , & Visconti, A. (2003). Determination of T‐2 toxin in cereal grains by liquid chromatography with fluorescence detection after immunoaffinity column clean‐up and derivatization with 1‐anthroylnitrile. Journal of Chromatography A, 989(2), 257–264. 10.1016/S0021-9673(03)00081-5 12650258

[fsn31474-bib-0111] Pedras, M. S. C. , Zaharia, L. I. , & Ward, D. E. (2002). The destruxins: Synthesis, biosynthesis, biotransformation, and biological activity. Phytochemistry, 59(6), 579–596. 10.1016/S0031-9422(02)00016-X 11867090

[fsn31474-bib-0112] Pennacchio, A. , Ruggiero, G. , Staiano, M. , Piccialli, G. , Oliviero, G. , Lewkowicz, A. , … D'Auria, S. (2014). A surface plasmon resonance based biochip for the detection of patulin toxin. Optical Materials, 36(10), 1670–1675. 10.1016/j.optmat.2013.12.045

[fsn31474-bib-0113] Pettersson, H. , & Langseth, W. (2002). Intercomparison of trichothecene analysis and feasibility to produce certified calibrants and reference material. Final report I, method studies. BCR Information, Project Report EUR 20285/1 EN 1–82. Brussels, Belgium: European Commission.

[fsn31474-bib-0114] Plattner, R. D. , & Shackelford, D. D. (1992). Biosynthesis of labeled fumonisins in liquid cultures of *Fusarium moniliforme* . Mycopathologia, 117(1–2), 17–22. 10.1007/BF00497274 1513369

[fsn31474-bib-0115] Puel, O. , Galtier, P. , & Oswald, I. (2010). Biosynthesis and toxicological effects of patulin. Toxins, 2(4), 613–631. 10.3390/toxins2040613 22069602 PMC3153204

[fsn31474-bib-0116] Radoi, A. , Targa, M. , Prieto‐Simon, B. , & Marty, J.‐L. (2008). Enzyme‐linked immunosorbent assay (ELISA) based on superparamagnetic nanoparticles for aflatoxin M1 detection. Talanta, 77(1), 138–143. 10.1016/j.talanta.2008.05.048 18804611

[fsn31474-bib-0117] Ran, C. , Chen, D. , Ma, H. , & Jiang, Y. (2017). Graphene oxide adsorbent based dispersive solid phase extraction coupled with multi-pretreatment clean-up for analysis of trace aflatoxins in traditional proprietary Chinese medicines. Journal of Chromatography B, 1044, 120–126. 10.1016/j.jchromb.2017.01.001 28092852

[fsn31474-bib-0118] Ren, M. , Xu, H. , Huang, X. , Kuang, M. , Xiong, Y. , Xu, H. , & Wang, A. (2014). Immunochromatographic assay for ultrasensitive detection of aflatoxin B1 in maize by highly luminescent quantum dot beads. ACS Applied Materials & Interfaces, 6(16), 14215–14222. 10.1021/am503517 25109633 PMC4149326

[fsn31474-bib-0119] Rheeder, J. P. , Marasas, W. F. , & Vismer, H. F. (2002). Production of fumonisin analogs by *Fusarium* species. Applied Environmental Microbiology, 68(5), 2101–2105. 10.1128/AEM.68.5.2101-2105.2002 11976077 PMC127586

[fsn31474-bib-0120] Righetti, L. , Bergmann, A. , Galaverna, G. , Rolfsson, O. , Paglia, G. , & Dall'Asta, C. (2018). Ion mobility‐derived collision cross section database: Application to mycotoxin analysis. Analytica Chimica Acta, 1014, 50–57. 10.1016/j.aca.2018.01.047 29523251

[fsn31474-bib-0121] Roda, A. , Mirasoli, M. , Guardigli, M. , Michelini, E. , Simoni, P. , & Magliulo, M. (2006). Development and validation of a sensitive and fast chemiluminescent enzyme immunoassay for the detection of genetically modified maize. Analytical and Bioanalytical Chemistry, 384(6), 1269–1275. 10.1007/s00216-006-0308-6 16491341

[fsn31474-bib-0122] Rodríguez‐Carrasco, Y. , Berrada, H. , Font, G. , & Mañes, J. (2012). Multi‐mycotoxin analysis in wheat semolina using an acetonitrile‐based extraction procedure and gas chromatography‐tandem mass spectrometry. Journal of Chromatography A, 1270, 28–40. 10.1016/j.chroma.2012.10.061 23182289

[fsn31474-bib-0123] Rodríguez‐Carrasco, Y. , Moltó, J. C. , Berrada, H. , & Mañes, J. (2014). A survey of trichothecenes, zearalenone and patulin in milled grain‐based products using GC–MS/MS. Food Chemistry, 146, 212–219. 10.1016/j.foodchem.2013.09.053 24176334

[fsn31474-bib-0124] Rodríguez‐Carrasco, Y. , Moltó, J. C. , Mañes, J. , & Berrada, H. (2014). Development of a GC–MS/MS strategy to determine 15 mycotoxins and metabolites in human urine. Talanta, 128, 125–131. 10.1016/j.talanta.2014.04.072 25059139

[fsn31474-bib-0125] Santini, A. , Ferracane, R. , Somma, M. C. , Aragón, A. , & Ritieni, A. (2009). Multitoxin extraction and detection of trichothecenes in cereals: An improved LC‐MS/MS approach. Journal of the Science of Food and Agriculture, 89(7), 1145–1153. 10.1002/jsfa.3564

[fsn31474-bib-0126] Sauceda‐Friebe, J. C. , Karsunke, X. Y. Z. , Vazac, S. , Biselli, S. , Niessner, R. , & Knopp, D. (2011). Regenerable immuno‐biochip for screening ochratoxin A in green coffee extract using an automated microarray chip reader with chemiluminescence detection. Analytica Chimica Acta, 689(2), 234–242. 10.1016/j.aca.2011.01.030 21397079

[fsn31474-bib-0127] Şenyuva, H. Z. , & Gilbert, J. (2010). Immunoaffinity column clean‐up techniques in food analysis: A review. Journal of Chromatography B, 878(2), 115–132. 10.1016/j.jchromb.2009.05.042 19525155

[fsn31474-bib-0128] Seo, J. A. , & Lee, Y. W. (1999). Natural occurrence of the C series of fumonisins in moldy corn. Applied and Environmental Microbiology, 65(3), 1331–1334. 10.1128/AEM.65.3.1331-1334.1999 10049903 PMC91184

[fsn31474-bib-0129] Shephard, G. S. , Sydenham, E. W. , Thiel, P. G. , & Gelderblom, W. C. A. (1990). Quantitative determination of fumonisins B1 and B2 by high-performance liquid chromatography with fluorescence detection. Journal of Liquid Chromatography, 13(10), 2077–2087.

[fsn31474-bib-0130] Simeon, N. , Myers, R. , Bayle, C. , Nertz, M. , Stewart, J. K. , & Couderc, F. (2001). Some applications of near‐ultraviolet laser‐induced fluorescence detection in nanomolar‐and subnanomolar‐range high‐performance liquid chromatography or micro‐high‐performance liquid chromatography. Journal of Chromatography A, 913(1–2), 253–259. 10.1016/S0021-9673(00)01234-6 11355820

[fsn31474-bib-0131] Smeesters, L. , Meulebroeck, W. , Raeymaekers, S. , & Thienpont, H. (2015). Optical detection of aflatoxins in maize using one‐and two‐photon induced fluorescence spectroscopy. Food Control, 51, 408–416. 10.1016/j.foodcont.2014.12.003

[fsn31474-bib-0132] Squires, T. M. , & Quake, S. R. (2005). Microfluidics: Fluid physics at the nanoliter scale. Reviews of Modern Physics, 77(3), 977–1026. 10.1103/RevModPhys.77.977

[fsn31474-bib-0133] Streit, E. , Schatzmayr, G. , Tassis, P. , Tzika, E. , Marin, D. , Taranu, I. , … Oswald, I. P. (2012). Current situation of mycotoxin contamination and co‐occurrence in animal feed‐Focus on Europe. Toxins, 4(10), 788–809. 10.3390/toxins4100788 23162698 PMC3496989

[fsn31474-bib-0134] Sudakin, D. L. (2003). Trichothecenes in the environment: Relevance to human health. Toxicology Letters, 143(2), 97–107. 10.1016/S0378-4274(03)00116-4 12749813

[fsn31474-bib-0136] Taheri, N. , Semnani, S. , Roshandel, G. , Namjoo, M. , Keshavarzian, H. , Chogan, A. G. , & Joshaghani, H. (2012). Aflatoxin contamination in wheat flour samples from Golestan Province, Northeast of Iran. Iranian Journal of Public Health, 41(9), 42. PMC3494214 23193505

[fsn31474-bib-0137] Tang, X. , Li, X. , Li, P. , Zhang, Q. I. , Li, R. , Zhang, W. , … Zhang, Z. (2014). Development and Application of an Immunoaffinity Column Enzyme Immunoassay for Mycotoxin Zearalenone in Complicated Samples. PLoS ONE, 9(1), e85606 10.1371/journal.pone.0085606 24465616 PMC3894983

[fsn31474-bib-0138] Tessini, C. , Mardones, C. , von Baer, D. , Vega, M. , Herlitz, E. , Saelzer, R. , … Torres, O. (2010). Alternatives for sample pre‐treatment and HPLC determination of Ochratoxin A in red wine using fluorescence detection. Analytica Chimica Acta, 660(1–2), 119–126. 10.1016/j.aca.2009.11.011 20103152

[fsn31474-bib-0139] Torres‐Pacheco, I. (Ed.) (2011). Aflatoxins: Detection, measurement and control. Norderstedt, Germany: BoD–Books on Demand.

[fsn31474-bib-0140] Trebstein, A. , Seefelder, W. , Lauber, U. , & Humpf, H. U. (2008). Determination of T‐2 and HT‐2 toxins in cereals including oats after immunoaffinity cleanup by liquid chromatography and fluorescence detection. Journal of Agricultural and Food Chemistry, 56(13), 4968–4975. 10.1021/jf800316m 18553918

[fsn31474-bib-0141] Tripathi, S. , & Mishra, H. N. (2009). A rapid FT‐NIR method for estimation of aflatoxin B1 in red chili powder. Food Control, 20(9), 840–846. 10.1016/j.foodcont.2008.11.003

[fsn31474-bib-0142] Trucksess, M. W. , & Tang, Y. I. F. E. N. G. (1999). Solid‐phase extraction method for patulin in apple juice and unfiltered apple juice. Journal AOAC International, 82, 1109–1114. 10513012

[fsn31474-bib-0143] Tsao, R. , & Zhou, T. (2000). Micellar electrokinetic capillary electrophoresis for rapid analysis of patulin in apple cider. Journal of Agricultural and Food Chemistry, 48(11), 5231–5235. 10.1021/jf000217c 11087465

[fsn31474-bib-0144] Turner, N. W. , Subrahmanyam, S. , & Piletsky, S. A. (2009). Analytical methods for determination of mycotoxins: A review. Analytica Chimica Acta, 632(2), 168–180. 10.1016/j.aca.2008.11.010 19110091

[fsn31474-bib-0145] Ueno, Y. (1984). Toxicological features of T‐2 toxin and related trichothecenes. Toxicological Sciences, 4(2Part2), 124–132. 10.1093/toxsci/4.2part2.124 6609858

[fsn31474-bib-0146] Ulbricht, M. , Matuschewski, H. , Oechel, A. , & Hicke, H. G. (1996). Photo‐induced graft polymerization surface modifications for the preparation of hydrophilic and low‐proten‐adsorbing ultrafiltration membranes. Journal of Membrane Science, 115(1), 31–47. 10.1016/0376-7388(95)00264-2

[fsn31474-bib-0147] Urraca, J. L. , Marazuela, M. D. , & Moreno‐Bondi, M. C. (2004). Analysis for zearalenone and α‐zearalenol in cereals and swine feed using accelerated solvent extraction and liquid chromatography with fluorescence detection. Analytica Chimica Acta, 524(1–2), 175–183. 10.1016/j.aca.2004.03.093

[fsn31474-bib-0148] Urry, W. H. , Wehrmeister, H. L. , Hodge, E. B. , & Hidy, P. H. (1966). The structure of zearalenone. Tetrahedron Letters, 7(27), 3109–3114. 10.1016/S0040-4039(01)99923-X

[fsn31474-bib-0149] Vail, R. B. , & Homann, M. J. (1990). Rapid and sensitive detection of citrinin production during fungal fermentation using high‐performance liquid chromatography. Journal of Chromatography A, 535, 317–323. 10.1016/S0021-9673(01)88958-5 2089062

[fsn31474-bib-0150] Vazquez, B. I. , Fente, C. , Franco, C. , Cepeda, A. , Prognon, P. , & Mahuzier, G. (1996). Simultaneous high‐performance liquid chromatographic determination of ochratoxin A and citrinin in cheese by time‐resolved luminescence using terbium. Journal of Chromatography A, 727(2), 185–193. 10.1016/0021-9673(95)01174-9

[fsn31474-bib-0151] Visconti, A. , Lattanzio, V. M. T. , Pascale, M. , & Haidukowski, M. (2005). Analysis of T‐2 and HT‐2 toxins in cereal grains by immunoaffinity clean‐up and liquid chromatography with fluorescence detection. Journal of Chromatography A, 1075(1–2), 151–158. 10.1016/j.chroma.2005.04.009 15974128

[fsn31474-bib-0152] Wang, X. , Liu, B. , Lu, Q. , & Qu, Q. (2014). Graphene‐based materials: Fabrication and application for adsorption in analytical chemistry. Journal of Chromatography A, 1362, 1–15. 10.1016/j.chroma.2014.08.023 25160951

[fsn31474-bib-0153] Wang, Y.‐K. , Wang, Y.‐C. , Wang, H.‐A. , Ji, W.‐H. , Sun, J.‐H. , & Yan, Y.‐X. (2014). An immunomagnetic‐bead‐based enzyme‐linked immunosorbent assay for sensitive quantification of fumonisin B1. Food Control, 40(1), 41–45. 10.1016/j.foodcont.2013.11.025

[fsn31474-bib-0154] Welke, J. E. , Hoeltz, M. , Dottori, H. A. , & Noll, I. B. (2009). Quantitative analysis of patulin in apple juice by thin‐layer chromatography using a charge coupled device detector. Food Additives & Contaminants. Part A, Chemistry, Analysis, Control, Exposure & Risk. Assessment, 26(5), 754–758. 10.1080/02652030802662746 19680947

[fsn31474-bib-0155] Woudenberg, J. H. C. , Groenewald, J. Z. , Binder, M. , & Crous, P. W. (2013). Alternaria redefined. Studies in Mycology, 75, 171–212. 10.3114/sim0015 24014900 PMC3713888

[fsn31474-bib-0156] Wu, Q. , & Xu, H. (2019). Application of multiplexing fiber optic laser induced fluorescence spectroscopy for detection of aflatoxin B1 contaminated pistachio kernels. Food Chemistry, 290, 24–31. 10.1016/j.foodchem.2019.03.079 31000043

[fsn31474-bib-0157] Xie, Y. J. , Yang, Y. , Kong, W. J. , Yang, S. H. , & Yang, M. H. (2015). Application of nanoparticle probe‐based lateral flow immunochromatographic assay in mycotoxins detection. Fenxi Huaxue/Chinese Journal of Analytical Chemistry, 43(4), 618–628. 10.1016/S1872-2040(15)60821-0

[fsn31474-bib-0158] Xing, Y. , Meng, W. , Sun, W. , Li, D. , Yu, Z. , Tong, L. , & Zhao, Y. (2016). Simultaneous qualitative and quantitative analysis of 21 mycotoxins in Radix Paeoniae Alba by ultra‐high performance liquid chromatography quadrupole linear ion trap mass spectrometry and QuEChERS for sample preparation. Journal of Chromatography B: Analytical Technologies in the Biomedical and Life Sciences, 1031, 202–213. 10.1016/j.jchromb.2016.07.008 27500642

[fsn31474-bib-0159] Xu, X. , Liu, X. , Li, Y. , & Ying, Y. (2013). A simple and rapid optical biosensor for detection of aflatoxin B1 based on competitive dispersion of gold nanorods. Biosensors and Bioelectronics, 47, 361–367. 10.1016/j.bios.2013.03.048 23603134

[fsn31474-bib-0160] Xu, Y. , Huang, Z. B. , He, Q. H. , Deng, S. Z. , Li, L. S. , & Li, Y. P. (2010). Development of an immunochromatographic strip test for the rapid detection of deoxynivalenol in wheat and maize. Food Chemistry, 119(2), 834–839. 10.1016/j.foodchem.2009.08.049

[fsn31474-bib-0161] Yang, M. , Sun, S. , Kostov, Y. , & Rasooly, A. (2011). An automated point‐of‐care system for immunodetection of staphylococcal enterotoxin B. Analytical Biochemistry, 416(1), 74–81. 10.1016/j.ab.2011.05.014 21640067 PMC3148523

[fsn31474-bib-0162] Yeh, C. H. , Chen, W. T. , Lin, H. P. , Chang, T. C. , & Lin, Y. C. (2009). Development of an immunoassay based on impedance measurements utilizing an antibody‐nanosilver probe, silver enhancement, and electro‐microchip. Sensors and Actuators B: Chemical, 139(2), 387–393. 10.1016/j.snb.2009.03.029

[fsn31474-bib-0163] Yu, L. L. , Wang, S. , & Sun, B. G. (2014). Food safety chemistry: Toxicant occurrence, analysis and mitigation. Boca Raton, FL: CRC Press.

[fsn31474-bib-0164] Yuan, J. , Deng, D. , Lauren, D. R. , Aguilar, M. I. , & Wu, Y. (2009). Surface plasmon resonance biosensor for the detection of ochratoxin A in cereals and beverages. Analytica Chimica Acta, 656(1–2), 63–71. 10.1016/j.aca.2009.10.003 19932816

[fsn31474-bib-0165] Zahn, M. , Jeong, M. L. , Wang, D. , Trinh, T. , Fay, B. , & Ma, W. (2009). Product‐specific sample clean‐up and HPLC analysis of aflatoxins for a dietary product. Phytochemical Analysis, 20(4), 335–337. 10.1002/pca.1132 19425113

[fsn31474-bib-0166] Zain, M. E. (2011). Impact of mycotoxins on humans and animals. Journal of Saudi Chemical Society, 15(2), 129–144. 10.1016/j.jscs.2010.06.006

[fsn31474-bib-0167] Zhang, Z. , Li, Y. , Li, P. , Zhang, Q. , Zhang, W. , Hu, X. , & Ding, X. (2014). Monoclonal antibody-quantum dots CdTe conjugate-based fluoroimmunoassay for the determination of aflatoxin B1 in peanuts. Food chemistry, 146, 314–319. 24176348 10.1016/j.foodchem.2013.09.048

[fsn31474-bib-0168] Zinedine, A. , Soriano, J. M. , Juan, C. , Mojemmi, B. , Moltó, J. C. , Bouklouze, A. , … Mañes, J. (2007). Incidence of ochratoxin A in rice and dried fruits from Rabat and Salé area, Morocco. Food Additives and Contaminants, 24(3), 285–291. 10.1080/02652030600967230 17364931

